# Dietary Acrylamide Exposure and Risk of Site-Specific Cancer: A Systematic Review and Dose-Response Meta-Analysis of Epidemiological Studies

**DOI:** 10.3389/fnut.2022.875607

**Published:** 2022-04-25

**Authors:** Tommaso Filippini, Thorhallur I. Halldorsson, Carolina Capitão, Raquel Martins, Konstantinos Giannakou, Janneke Hogervorst, Marco Vinceti, Agneta Åkesson, Karin Leander, Andromachi Katsonouri, Osvaldo Santos, Ana Virgolino, Federica Laguzzi

**Affiliations:** ^1^Environmental, Genetic and Nutritional Epidemiology Research Center (CREAGEN)–Section of Public Health, Department of Biomedical, Metabolic and Neural Sciences, University of Modena and Reggio Emilia, Modena, Italy; ^2^Department of Epidemiology Research, Centre for Fetal Programming, Copenhagen, Denmark; ^3^Faculty of Food Science and Nutrition, University of Iceland, Reykjavík, Iceland; ^4^EnviHeB Lab, Faculdade de Medicina, Instituto de Saúde Ambiental, Universidade de Lisboa, Lisbon, Portugal; ^5^Laboratório Associado TERRA, Faculdade de Medicina, Universidade de Lisboa, Lisbon, Portugal; ^6^State General Laboratory, Ministry of Health, Nicosia, Cyprus; ^7^Department of Health Sciences, School of Sciences, European University Cyprus, Nicosia, Cyprus; ^8^Centre for Environmental Sciences, Hasselt University, Diepenbeek, Belgium; ^9^Department of Epidemiology, Boston University School of Public Health, Boston, MA, United States; ^10^Unit of Cardiovascular and Nutritional Epidemiology, Institute of Environmental Medicine, Karolinska Institute, Stockholm, Sweden; ^11^Unbreakable Idea Research, Cadaval, Portugal

**Keywords:** acrylamide, dietary exposure, neoplasms, carcinoma, systematic review, meta-analysis, epidemiological studies

## Abstract

Diet is a main source of acrylamide exposure to humans. Existing observational data on the relationship between dietary exposure to acrylamide and risk of cancer are inconsistent. We performed a systematic review and dose-response meta-analysis of epidemiological studies evaluating the association between dietary acrylamide exposure and several site-specific cancer. A systematic literature search was conducted in PubMed, Scopus, and Web of Science databases until March 7, 2022. Studies were eligible if they were carried out in non-occupationally exposed adults, assessed dietary acrylamide exposure (μg/day) and reported risk estimates of cancer incidence (all but gynecological cancers). Using a random-effects model, we performed a meta-analysis of site-specific cancer risk comparing the highest vs. lowest category of dietary acrylamide exposure. We also carried out a one-stage dose-response meta-analysis assessing the shape of the association. Out of 1,994 papers screened, 31 were eligible (total of 16 studies), which included 1,151,189 participants in total, out of whom 48,175 developed cancer during the median follow-up period of 14.9 years (range 7.3–33.9). The mean estimated dose of dietary acrylamide across studies was 23 μg/day. Pooled analysis showed no association between the highest vs. lowest dietary acrylamide exposure and each site-specific cancer investigated, with no evidence of thresholds in the dose-response meta-analysis. There were also no associations between dietary acrylamide exposure and the risk of cancers when stratifying by smoking status, except for increased risk of lung cancer in smokers. In conclusion, high dietary acrylamide exposure was not associated with an increased risk of site-specific non-gynecological cancer.

## Introduction

Acrylamide is a chemical compound mainly used in industry to produce polyacrylamides that are employed for different purposes such as flocculants, dyes, paper, and textiles. Beside the potential exposure of acrylamide in the occupational setting, the main sources of exposure to this substance in the general population is through the intake of certain foods, high in carbohydrates cooked at temperatures >120°C, especially in low moisture conditions (1). Acrylamide may also be formed in foods from acrolein, a compound produced during the degradation of amino acids, carbohydrates, lipids, and organic acids (2). The content of acrylamide in foods varies largely, depending on the food matrix and the food processing method. Thus, dietary exposure to acrylamide differs across countries and global geographical areas, due to distinct traditional diets and its culinary habits (3). Tobacco smoke is also a source of acrylamide, making smokers potentially exposed to higher levels of acrylamide compared to non-smokers (4, 5). Smokers have been shown to have, on average, three to four times higher levels of acrylamide biomarkers (i.e., hemoglobin adducts) compared to non-smokers (6). Additional sources of exposure to acrylamide take place in occupational settings, where an association to neurological alterations has been shown (7).

Acrylamide is classified as probably carcinogenic to humans (class 2A) by the International Agency for Research on Cancer (IARC) (8). However, the underlying mechanisms of the carcinogenicity of acrylamide are far from being understood. Still, experimental evidence has shown that the main metabolite of acrylamide, glycidamide, is genotoxic—highly reactive toward DNA and proteins (9, 10). A study found a glycidamide-related mutational signature in one-third of approximately 1,600 human tumor genomes corresponding to 19 types of cancer (11). Non-genotoxic modes of action of acrylamide involving, for instance, hormonal pathways, are also proposed as underlying mechanisms of carcinogenicity, driving cell transformation or proliferation and apoptosis, independently of acrylamide-induced genotoxicity (9).

Although *in vitro* and animal studies have consistently shown that acrylamide is genotoxic, mutagenic, and carcinogenic (9, 12), epidemiological studies investigating the relationship between high dietary acrylamide exposure and the risk of several site-specific cancer (e.g., prostate, gastrointestinal and lung) in the general population and in occupational settings have reported inconsistent results, with some studies pointing to an increased risk (13–19) and others to null association (3, 17, 18, 20–41). All of these studies used a dietary assessment method to estimate dietary acrylamide exposure except for one study performed in a Swedish population where acrylamide exposure was assessed through both dietary intake and hemoglobin adducts (20). So far, a few systematic reviews and meta-analyses based on epidemiological studies were performed to synthesize the body of evidence in this field (42–48). In the most recent and comprehensive meta-analysis published in 2015, no association between high dietary acrylamide exposure and the risk of several cancers was noted, except for a modest increased risk of renal cancer (46), which was not observed in another meta-analysis investigating dietary acrylamide exposure and renal cell carcinoma (44). With the exception of a recent study investigating the relationship between dietary acrylamide exposure and female reproductive cancers, observing a relatively linear increased risk for ovarian and endometrial cancer (42), no dose-response meta-analysis exist for other cancer forms.

Hence, we aimed to synthesize the results on the association between dietary acrylamide exposure and risk of site-specific cancer. For this purpose, we performed a systematic literature review and dose-response meta-analysis of epidemiological studies evaluating the association between acrylamide and the risk of several site-specific cancer.

## Methods

### Literature Search

We performed a systematic literature review following the Preferred Reporting Items for Systematic Reviews and Meta-Analyses (PRISMA) (49) and the Meta-analysis Of Observational Studies in Epidemiology (MOOSE) guidelines (50). Two researchers (C.C. and R.M.) independently searched PubMed, Scopus, and Web of Science databases. A pairwise combination of two sets of terms [medical subject headings (MeSH) terms, whenever possible] were used, being then adapted for each database. One set included terms regarding the exposure to acrylamide and the other to the health outcomes under study. The protocol with the detailed search strategy used for each database, including the PICOS tool can be found in Supplementary Table 1. The literature searches were restricted to English language publications with no time limitation (up till the moment of the search, on March 7, 2022), or any other filter. An additional manual search was performed by screening the references of the included papers of individual studies.

### Study Selection

#### Inclusion Criteria

Prospective and retrospective cohort, case-cohort and case-control studies performed in non-occupationally exposed adults (≥18 years) with acrylamide exposure assessed through diet were considered for inclusion. The included papers reported risk estimates [risk ratio (RR), hazard ratio (HR) or odds ratio (OR)] with corresponding 95% confidence intervals (CIs) for any type of cancer in relation to dietary acrylamide exposure, except for the female reproductive cancers (see section “Exclusion Criteria”). Regarding exposure, eligible studies reported acrylamide exposure as continuous and/or categories (i.e., quintiles, quartiles, or tertiles). We considered for inclusion studies reporting number of cases, participants, or person-years per dietary acrylamide category. Studies were included when estimates were adjusted at least for smoking status since smoking has been observed to increase the level of acrylamide, could covary with the consumption of foods high in acrylamide, and is a risk factor for many of the cancer forms under study.

#### Exclusion Criteria

Studies investigating breast, endometrial or ovarian cancers were excluded, as these outcomes were part of a recently published dose-response meta-analysis (42).

#### Eligibility Assessment

All references resulting from the search were downloaded and duplicates were removed. Titles and abstracts retrieved were independently screened for eligibility by two teams of two authors (T.I.H., C.C., R.M., and A.V.). Disagreements were solved by a third author (T.F.). In case of missing information for the meta-analysis, the corresponding author of the paper was contacted for clarification. If no additional information was gathered, the paper was excluded.

### Data Extraction

Data from each selected paper were extracted by one of the research team members and independently checked by a second one (T.F., C.C., R.M., A.V., and F.L.). The following data were extracted: author, year of publication, type of study design, country including the geographical area, year of the baseline assessment, follow-up time, age, sex, smoking status, doses of acrylamide exposure (mean and/or median; according to what was available), number of cases, number of participants, person-years, risk estimates, and list of the confounders which were adjusted for. The risk estimates with their 95% confidence intervals were extracted from the model adjusted for smoking and the greatest number of other covariables.

The median or mean diet acrylamide exposure in each exposure category was assigned to the corresponding relative risk (RR), hazard ratio (HR), or odds ratio (OR). If the average intake in each category of exposure was not reported, the midpoint of the upper and lower boundaries of the category was assigned as the average intake. If the upper bound of the highest category or the lowest bound of the lowest category were not reported, they have been estimated using minus/plus 20% of the lower or higher open boundaries, respectively. For the RR reported for each quintile but with no respective intakes reported, the values were calculated under the assumption of a normal distribution using the mean, standard deviation, and zeta for each quintile considered (X-SD*Z quintile).

Any site-specific cancer (except for female reproductive cancers) previously investigated in relation to dietary acrylamide exposure was included in the analysis.

### Risk of Bias Assessment

The risk of bias was assessed by C.C., R.M., and A.V. using the Cochrane Collaboration tool for assessing risk of bias in non-randomized studies of interventions (ROBINS-I) (51). Risk of bias for each of the six ROBINS-I domains was classified as low, moderate, serious, critical or with lack of information (“no information”). The assessment within each of the six domains was used to determine an overall risk of bias for the outcome under assessment.

### Data Analysis

We employed a restricted maximum likelihood random-effects meta-analysis to assess summary RR along with 95% confidence interval (CI) for high dietary acrylamide exposure in comparison to low dietary exposure for each site-specific cancer, when at least two risk estimates were available on the same outcome.

A dose-response meta-analysis of the association between dietary acrylamide exposure and site-specific cancer was performed using the one-stage approach recently employed in other research fields (52, 53). Potential non-linear associations were evaluated using cubic splines with knots at 3 fixed points (10th, 50th, and 90th percentiles) of the exposure through a multivariate random-effects meta-analysis using the restricted maximum likelihood method (54). The reference value for the dose-response meta-analysis was set at the mean value of all the included studies, i.e., 23 μg/day. We also fitted a linear regression model reporting its slope alongside the non-linear relationship yielded by the spline analysis.

Additional analyses were conducted stratifying by geographical area (West and East) due to potential differences in the dietary exposure to acrylamide across countries (3), and smoking status since smokers have been shown to have higher levels of acrylamide than never smokers (55).

We assessed the possible presence of publication bias and small-study bias using funnel plots and Egger’s regression asymmetry test for studies reporting highest vs. lowest exposure when at least five studies evaluated the same outcome (56, 57). We also assessed heterogeneity using the *I*^2^ statistic, and through the evaluation of the effect of variation across studies using a graphical overlay of study-specific predicted curves (58). All statistical analyses were conducted with STATA, version 17.0 (Stata Corp., College Station, TX, United States, 2021).

## Results

**FIGURE 1:**
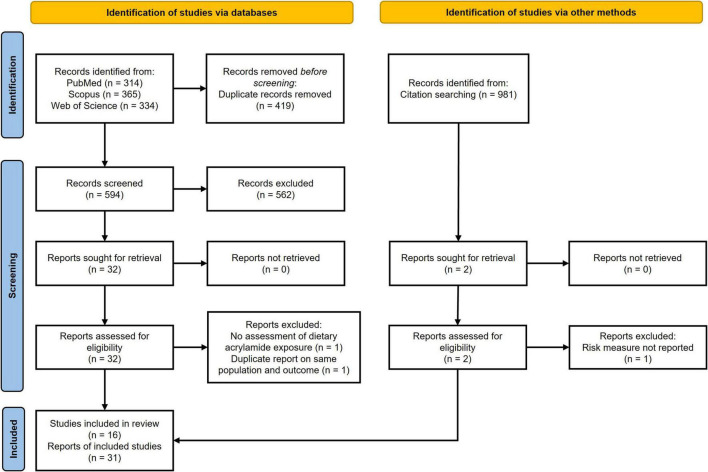
Flow chart of the included study following PRISMA guidelines.

Out of 1,994 papers retrieved through the literature and manual search, we identified 31 papers that met the inclusion criteria, corresponding to a total of 16 studies. Detailed information on the selection of the studies included is presented in Figure 1. Descriptive characteristics of the studies included are presented in Table 1. Most studies were performed in Europe (*n* = 21), primarily in the Netherlands (n = 8) and Sweden (*n* = 7), followed by Japan (*n* = 6) and the United States (*n* = 3). A total of 1,151,189 participants were included in these case-control, case-cohort, and cohort studies. Participants’ age ranged from 50 to 70 years (mean: 60 years). During a median follow-up period of 14.9 years (range: 7.3–33.9 years), 48,175 participants developed cancer. The following site-specific cancer were investigated in the papers in relation to dietary acrylamide exposure: oral cavity, esophageal, stomach, colorectal (including colon and rectal), pancreatic, laryngeal, lung, lymphoma, multiple myeloma, renal, bladder/urothelial, prostate, melanoma, and brain. Dietary acrylamide was assessed through food frequency questionnaires in all the analyzed studies. The mean and median estimated dose of dietary acrylamide across studies was 23 μg/day.

**TABLE 1:** Descriptive characteristics of the included studies by site-specific cancer.

References	Study design	Country	Baseline (Follow-up median years)	Total participants	Cases	Acrylamide dose (μ g/day) median (high vs. low)^a^	Subgroups analyzed (notes)
**Oral cavity**							
Pelucchi et al. (22)	Case-control	Italy; Switzerland	1991–1997	2,521	749	27.3 (48.5; 10.3)	Sex and never smoking
Schouten et al. (31)	Case-cohort	The Netherlands	1986 (16.3)	4,212	101	21.8 (37.2; 10.1)	Sex and never smokers
**Oro and hypopharynx**							
Schouten et al. (31)	Case-cohort	The Netherlands	1986 (16.3)	4,194	83	21.8 (37.2; 10.1)	Sex and never smokers
**Esophageal**							
Hogervorst et al. (17)	Case-cohort	The Netherlands	1986 (13.3)	4,654	216	21.8 (37.3;6.3)	Never smokers
Lin et al. (14)	Case-control	Sweden	1995–1997	1,414	594	36.3 (52.9; 21.8)	
Liu et al. (29)	Cohort	Japan	1990;1993 (15.5)	87,628	391	6.8 (12.7; 2.8)	
Lujan-Barroso et al. (32)	Cohort	10 European countries*	1992–1998 (11)	477,308	341	23.3 (37.9; 11.3)	Never smokers
Pelucchi et al. (22)	Case-control	Italy; Switzerland	1992–1999	1,461	395	26.9 (47.5; 10.6)	
**Stomach**							
Hirvonen et al. (60)	Cohort	Finland	1985–1988 (10.2)	27,111	224	36.8 (55.7; 21.9)	(Only smokers men)
Hogervorst et al. (17)	Case-cohort	The Netherlands	1986 (13.3)	5,001	563	21.8 (37.3; 6.3)	Never smokers
Liu et al. (29)	Cohort	Japan	1990;1993 (15.3)	87,628	2,218	6.8 (12.7; 2.8)	
**Colorectal**							
Hirvonen et al. (60)	Cohort	Finland	1985–1988 (10.2)	27,111	316	36.8 (55.7; 21.9)	(Only smokers men)
Hogervorst et al. (17)	Case-cohort	The Netherlands	1986 (13.3)	6,628	2,190	21.8 (37.3; 6.3)	Never smokers
Larsson et al. (28)	Cohort	Sweden	1997 (9.3)	45,306	676	36.2 (50.0; 33.7)	(Only men)
Liu et al. (29)	Cohort	Japan	1990–1993 (15.3)	87,628	2,470	6.8 (12.7; 2.8)	
Mucci et al. (21)	Case-control	Sweden	1992–1994	1,129	591	24.0 (45.4; 11.8)	Never smokers
Mucci et al. (33)	Cohort	Sweden	1987–1990 (16)	61,467	446	24.7 (37.9; 12.8)	Only women
Pelucchi et al. (22)	Case-control	Italy; Switzerland	1992–2001	7,045	2,280	26.6 (47.6; 10.0)	
**Colon**							
Hogervorst et al. (17)	Case-cohort	The Netherlands	1986 (13.3)	5,943	1,505	21.8 (37.3; 6.3)	Never smokers
Larsson et al. (28)	Cohort	Sweden	1997 (9.3)	45,306	410	36.2 (50.0; 33.7)	(Only men)
Liu et al. (29)	Cohort	Japan	1990;1993 (15.3)	87,628	1,721	6.8 (12.7; 2.8)	
Mucci et al. (33)	Cohort	Sweden	1987–1990 (16)	61,467	307	24.7 (37.9; 12.8)	(Only women)
**Rectal**							
Hogervorst et al. (17)	Case-cohort	The Netherlands	1986 (13.3)	4,948	510	21.8 (37.3; 6.3)	Never smokers
Larsson et al. (28)	Cohort	Sweden	1997 (9.3)	45,306	266	36.2 (50.0; 33.7)	(Only men)
Liu et al. (29)	Cohort	Japan	1990;1993 (15.3)	87,628	749	6.8 (12.7; 2.8)	
Mucci et al. (33)	Cohort	Sweden	1987–1990 (16)	61,467	144	24.7 (37.9; 12.8)	(Only women)
**Pancreatic**							
Hirvonen et al. (60)	Cohort	Finland	1985–1988 (10.2)	27,111	192	36.8 (55.7; 21.9)	(Only smokers men)
Hogervorst et al. (17)	Case-cohort	The Netherlands	1986 (13.3)	4,787	349	21.8 (37.3; 6.3)	Never smokers
Kito et al. (71)	Cohort	Japan	1990;1993 (15.2)	89,728	576	6.9 (11.0; 3.3)	Sex and smoking
Obón-Santacana (72)	Cohort	10 European countries*	1992–1998 (11)	477,308	865	26.2 (44.5; 11.3)	Sex and smoking
Pelucchi et al. (23)	Case-control	Italy	1991–2008	978	326	32.6 (53.7; 13.0)	
Pelucchi et al. (25)	Case-control	United States, Italy, Austria		3,130	895	22.8 (34.8; 10.9)	
**Liver**							
Zha et al. (35)	Cohort	Japan	1995–1998 (16)	85,305	744	6.9 (11.1; 3.4)	Sex and smoking
**Head and neck**							
Schouten et al. (31)	Case-cohort	The Netherlands	1986 (16.3)	4,468	357	21.8 (37.2; 10.1)	Sex and never smokers
**Larynx**							
Schouten et al. (31)	Case-cohort	The Netherlands	1986 (16.3)	4,291	180	21.8 (37.2; 10.1)	Sex and never smokers
Pelucchi et al. (22)	Case-control	Italy; Switzerland		1,824	527	26.4 (45.8; 10.5)	Sex and smoking
**Lung**							
Hirvonen et al. (60)	Cohort	Finland	1985–1988 (10.2)	27,111	1,703	36.8 (55.7; 21.9)	(Only smokers men)
Hogervorst et al. (19) (F; M)	Case-cohort	The Netherlands	1986 (13.3)	120,852	2,649	21.6 F (36.8; 9.5)-22.6 M (37.6; 10.8)	Never smoking
Liu et al. (3) (F; M)	Cohort	Japan	1990; 1993 (14.8)	85,303	1,187	6.8 F (12.0; 3.2)-7.0 M (12.1; 2.9)	Smoking
**Thyroid**							
Schouten et al. (31)	Case-cohort	The Netherlands	1986 (16.3)	4,177	66	21.8 (32.5;12.0)	Female and never smokers
**Bladder**							
Hirvonen et al. (60)	Cohort	Finland	1985–1988 (10.2)	27,111	365	36.8 (55.7; 21.9)	(Only smokers men)
Hogervorst et al. (17)	Case-cohort	The Netherlands	1986 (13.3)	3,401	1,210	22.6 (38.2; 7.0)	Never smokers
Ikeda et al. (59)	Cohort	Japan	1990;1993 (15.5)	88,818	392	7.1 (11.2; 3.6)	Heterogeneity for smokers
Mucci et al. (21)	Case-control	Sweden	1992–1994	801	263	24.7 (46.3; 12.5)	Never smokers
**Renal**							
Hirvonen et al. (60)	Cohort	Finland	1985–1988 (10.2)	27,111	184	36.8 (55.7; 21.9)	(Only smokers men)
Hogervorst et al. (17)	Case-cohort	The Netherlands	1986 (13.3)	2,530	339	22.6 (38.2; 7.0)	Smokers
Graff et al. (30) (F; M)	Cohort	United States	1986 (27.2)	136,564	629	15.8 F (25.8; 7.1)-21.7 M (35.1; 11.1)	Never smoking
Ikeda et al. (59)	Cohort	Japan	1990;1993 (15.5)	88,818	208	7.06 (11.2; 3.6)	Smoking
McCullough et al. (73)	Cohort	United States	1999 (14)	102,154	412	22.55 (33.0; 13.4)	Sex and never smokers
Mucci et al. (21)	Case-control	Sweden	1992–1994	671	133	24.0 (44.3; 12.5)	Never smokers
Mucci et al. (74)	Case-control	Sweden	1987	722	376	26.57 (38.3; 16.1)	Sex and smokers
Pelucchi et al. (36)	Case-control	Italy; Switzerland	1992–2004	1,534	767	38.3 (42.9; 16.3)	
**Prostate**							
Hirvonen et al. (60)	Cohort	Finland	1985–1988 (10.2)	27,111	799	36.8 (55.7; 21.9)	(Only smokers men)
Hogervorst et al. (17)	Case-cohort	The Netherlands	1986 (13.3)	4,437	2,246	22.6 (38.2; 7.0)	Never smokers
Ikeda et al. (60)	Cohort	Japan	1990;1993 (15.2)	88,818	1,195	7.1 (11.2; 3.6)	Smoking
Larsson et al. (27)	Cohort	Sweden	1997 (9.1)	45,306	610	36.3 (52.1; 22.6)	Never smokers
Pelucchi et al. (22)	Case-control	Italy and Switzerland	1992–2001	2,745	1,294	25.23 (43.6; 9.9)	
Perloy et al. (34)	Case-cohort	The Netherlands	1986 (20.3)	2,411	190	22.4 (37.7; 7.1)	Smoking
Wilson et al. (20)	Case-control	Sweden	2001–2002	2,504	1,489	44.10 (67.2; 26.4)	
Wilson et al. (24)	Cohort	United States	1986 (20)	47,896	5,025	22.2 (35.0; 12.0)	Never smokers
**Lymphoma**							
Bongers et al. (16) (F; M)	Case-cohort	The Netherlands	1986 (16.3)	5,348	910	21.0 F (36.4; 5.6)-23.0 M (38.4; 7.6)	Sex, never smokers, subtypes of lymphoma
Hirvonen et al. (60)	Cohort	Finland	1985–1988 (10.2)	27,111	175	36.8 (55.7; 21.9)	(Only smokers men)
Zha et al. (75)	Cohort	Japan	1990&1993 (16)	85,303	326	6.9 (11.1; 3.6)	Smoking
**Multiple myeloma**							
Bongers et al. (16) (F; M)	Case-cohort	The Netherlands	1986 (16.3)	4,761	323	21.0 F (36.4; 5.6)-23.0 M (38.4; 7.6)	Sex, never smokers and subtypes of multiple myeloma
Zha et al. (75)	Cohort	Japan	1990–1993 (16)	85,303	126	6.9 (11.1; 3.6)	Smoking
**Melanoma**							
Lipunova et al. (13) (F; M)	Case-cohort	The Netherlands	1986 (17.3)	5,134	224	21.1 F (36.8; 9.5)-22.6 M (37.6; 10.8)	Sex and never smokers (subtypes of melanoma)
**Brain**							
Hogervorst et al. (19)	Case-cohort	The Netherlands	1986 (16.3)	4,654	216	21.8 (37.3; 6.3)	Never smokers

*France, Italy, Spain, United Kingdom, The Netherlands, Greece, Germany, Sweden, Denmark, Norway. ^a^Estimated doses of acrylamide in high category vs. low reported for the overall population F, female; M, Males.

Summary estimates for the association between highest vs. lowest dietary acrylamide exposure and the risk of various types of cancer in smokers and never smokers combined, and by smoking status, are shown in Table 2 and in forest plots (Supplementary Figures 1–15). Regardless smoking status, summary estimates showed no association between higher dietary acrylamide and any of the site-specific cancer investigated.

**TABLE 2:** Summary relative risk (RR) with 95% confidence interval (CI) of the association between high dietary acrylamide exposure and site-specific cancer.

			All				Never smoker				Ever-smoker	

Type of cancer	N	RR	(95% CI)	I^2^	N	RR	(95% CI)	I^2^	N	RR	(95% CI)	I^2^
Oral cavity	2	0.99	(0.67–1.46)	16.9	1	1.06	(0.84–1.33)	–	–			
Oro and hypopharynx	1	0.61	(0.33–1.12)	–	–	–		–	–			
Esophageal	5	1.05	(0.85–1.29)	25.5	3	1.22	(0.77–1.95)	36.2	–			
Stomach	2	0.92	(0.82–1.05)	0.0	1	1.43	(0.92–2.23)	–	1	0.96	(0.60–1.53)	–
Colorectal	7	0.94	(0.87–1.02)	0.0	2	0.89	(0.46–1.73)	74.6	2	0.90	(0.64–1.26)	0.0
Colon	4	0.96	(0.85–1.09)	15.9	1	1.21	(0.86–1.70)	–	–			
Rectal	4	0.99	(0.84–1.18)	0.0	1	1.48	(0.77–2.84)	–	–			
Pancreatic	5	0.88	(0.77–1.02)	0.0	4	0.90	(0.74–1.10)	2.9	4	0.82	(0.65–1.03)	0.0
Liver	1	1.08	(0.87–1.34)	–	1	1.15	(0.85–1.56)	–	1	1.07	(0.80–1.43)	–
Head and neck	1	0.74	(0.50–1.09)	–	–	–			–	–		
Laryngeal	2	1.10	(0.79–1.54)	0.0	1	0.82	(0.53–1.28)	–	–			
Lung	2	0.91	(0.64–1.28)	83.1	2	0.92	(0.73–1.17)	0.0	2	1.16	(1.03–1.31)	0.0
Thyroid	1	1.33	(0.70–2.53)	-	1	1.03	(0.84–1.26)	–	–			
Bladder/Urothelial	3	0.89	(0.74–1.07)	0.0	3	0.85	(0.45–1.59)	72.5	3	0.94	(0.74–1.21)	0.0
Renal	7	1.08	(0.93–1.26)	19.4	4	1.13	(0.87–1.45)	0.0	3	1.10	(0.74–1.64)	0.0
Prostatic	7	1.00	(0.93–1.07)	0.0	4	0.98	(0.88–1.10)	0.0	4	0.96	(0.83–1.10)	0.0
Lymphoma	2	1.08	(0.95–1.22)	11.5	2	0.99	(0.68–1.44)	45.6	2	1.14	(0.81–1.62)	0.0
Multiple myeloma	2	0.97	(0.58–1.64)	63.6	2	0.98	(0.49–1.96)	50.5	1	0.52	(0.21–1.28)	–
Melanoma	1	1.18	(0.72–1.96)	–	1	1.14	(0.75–1.75)	–	–			
Brain	1	0.87	(0.54–1.41)	–	1	0.87	(0.46–1.64)	–	–			

*Results are shown for all subjects and for never smokers and ever smokers separately.*

**FIGURE 2:**
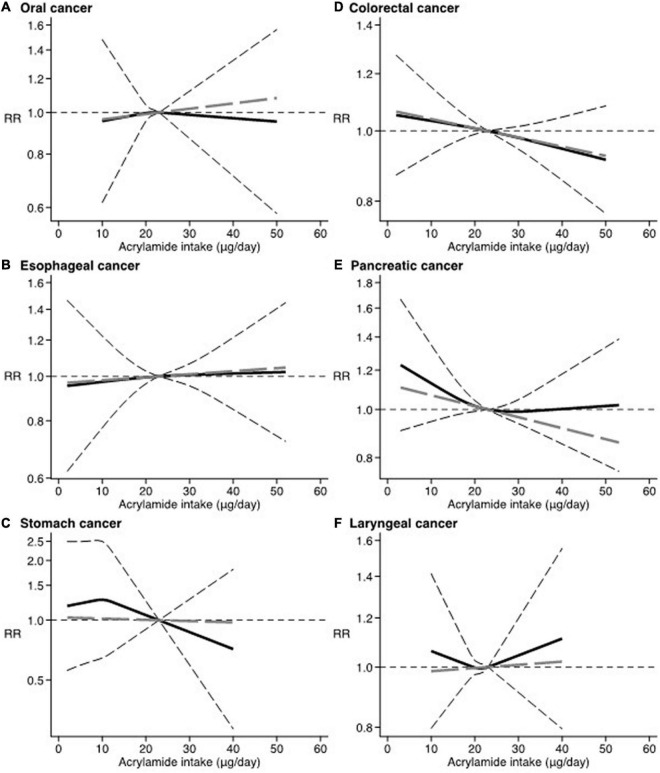
Dose-response. Spline curve (black solid line) with 95% confidence limits (black dashed lines). Linear trend (gray long-dashed line). RR, relative risk. Reference value of dietary acrylamide exposure: 23 μg/day. **(A)** Oral cancer (22, 31). **(B)** Esophageal cancer (14, 17, 22, 29, 32). **(C)** Stomach cancer (17, 29). **(D)** Colorectal cancer (17, 21, 22, 28, 29, 33). **(E)** Pancreatic cancer (17, 23, 71, 72). **(F)** Laryngeal cancer (22, 31).

**FIGURE 3:**
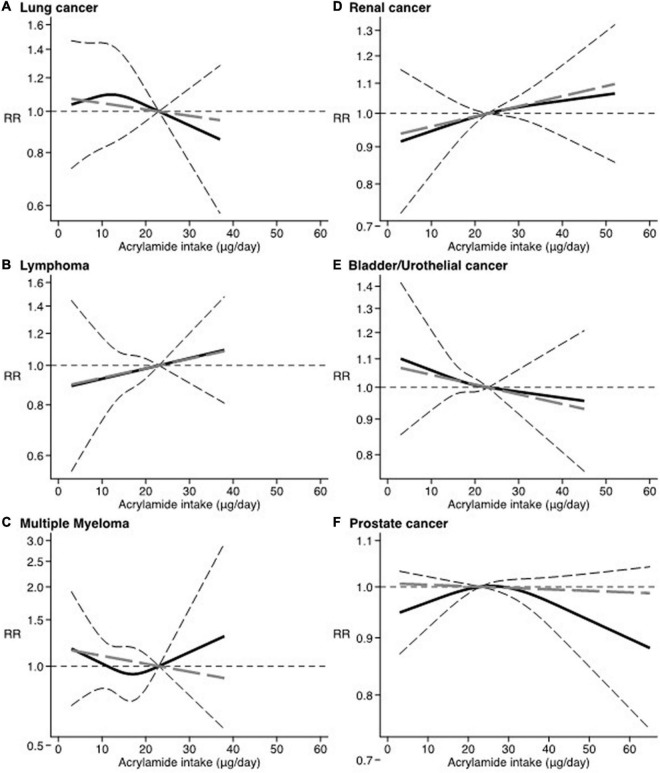
Dose-response. Spline curve (black solid line) with 95% confidence limits (black dashed lines). Linear trend (gray long-dashed line). RR: relative risk. Reference value of dietary acrylamide exposure: 23 μg/day. **(A)** Lung cancer (3, 19). **(B)** Lymphoma (16, 75). **(C)** Multiple myeloma (16, 75). **(D)** Renal cancer (17, 21, 30, 36, 59, 73, 74). **(E)** Bladder/Urothelial cancer (17, 21, 59). **(F)** Prostate cancer (17, 20, 22, 24, 27, 34, 59).

Results from the dose-response analysis showed a null association with no thresholds for all site-specific cancer considered in relation to increasing levels of dietary acrylamide (Figures 2, 3).

There was also no association between dietary acrylamide and the risk of site-specific cancer in the subgroups of smokers and never smokers, except for lung cancer, where we observed an increased risk in smokers (RR 1.16; 95% CI 1.03–1.31). Compared to smokers, the summary estimates for never smokers were observed to be higher, albeit very imprecise, for stomach, pancreatic, liver, renal, prostate, and multiple myeloma (Supplementary Figures 3, 7, 11, 12, 14).

Additional analysis by geographical regions (West and East) could be performed only excluding Asian population (i.e., Japan studies) since most of the studies were performed in the West area (Europe and United States). We observed a slightly, though imprecise, increased risk of lymphoma in relation to high dietary acrylamide when we consider only study from the West (*n* = 1) (RR 1.12; 95% CI 0.99–1.22) (Supplementary Figure 13).

There was no evidence of between-study heterogeneity except for lung and multiple myeloma cancers (Supplementary Figures 9, 14, respectively) (*I*^2^ > 50%). The graphical overlay of single study effects in the dose-response meta-analysis did not show substantial variation (data not shown). No evidence of publication bias, visualized by funnel plots, was observed (Supplementary Figures 2, 4, 7, 11, 12). The results of the risk of bias assessment, using the ROBINS-I are shown in Supplementary Figures 16, 17. Within the six dimensions analyzed, all studies had a low risk of bias regarding the measurement of outcomes and the reported result, and 75% of the studies showed a low risk of bias on the dimensions selection of participants and classification of interventions. All studies had a moderate risk of bias for confounding. There was missing information (classified as “no information”) regarding the dimensions’ deviations from intended interventions and missing data for more than 75% of the analyzed studies.

## Discussion

In this up-to-date systematic review and meta-analysis, based on 31 papers, we observed no association between high dietary acrylamide exposure and increased risk of any of the investigated cancers, including those of oral cavity, esophageal, gastric, colon-rectal, pancreatic, prostate, bladder, lung, renal, lymphoma, myeloma, thyroid, brain, larynx, and melanoma. As a novel finding, we found no thresholds between different levels of dietary acrylamide and the risk of any of the site-specific cancers considered. Considering studies performed in Western geographical areas alone, a slightly increased risk of lymphoma was observed in relation to high dietary acrylamide exposure. In general, findings did not differ by smoking status, except for increased risk of lung cancer in smokers.

Overall, our results of no association between high dietary acrylamide exposure and different site-specific cancers in the general population confirm the findings of previous meta-analyses that did not perform dose-response meta-analysis (22, 44, 46, 48). On the other hand, Pelucchi et al. (46) found a slight increased risk of renal cell carcinoma by 20% in relation to high dietary acrylamide (RR 1.20; 95% CI 1.00–1.45). This difference may be explained by the fact that we included three recent cohort studies with American (18, 30) and Japanese (59) participants. Results from these additional studies were to some extent heterogenous, with either a tendency of increased (18, 30) or decreased risk (30, 59) of renal cell carcinoma. The studies with American participants (18, 30) also contributed largely to the analysis (around 10% weight), possibly affecting our overall results. Moreover, we did not include the Finnish Alpha-Tocopherol, Beta-Carotene Cancer (ATBC) prevention study (60), a cohort study of 27,111 smoking men, in our main analysis which was based on both smokers and never smokers.

To the best of our knowledge, our meta-analysis is the first one investigating the shape of the dose-response relationships between dietary acrylamide exposure and the risk of site-specific cancer. Our results suggest that these associations, if present, may generally be without thresholds. These results are in line with those reported in a previous meta-analysis investigating the association between dietary acrylamide exposure and female reproductive cancers, which were not considered for inclusion in our meta-analysis (42).

Our aforementioned results may be explained by the relatively low dietary acrylamide exposure in the general population (intake ranged from 6.8 to 44.1 μg/day in the included studies—corresponding up to about 0.7 μg/kg body weight/day) when compared to the levels of acrylamide observed to be toxic in animal studies (50 mg/kg body weight/day) (9). There is also evidence showing that acrylamide does not generate any toxicologically detrimental effects when male rats were administrated three low oral doses of acrylamide (20, 40, and 90 μg/kg body weight/day, respectively) (61). Also, it is relevant to take into account that the EFSA CONTAM Panel selected BMDL_10_ value of 0.17 mg/kg body weight/day for neoplastic effects in mice (12), i.e., much higher level compared with those generally experienced by humans. Most of the included epidemiological studies investigating the relationship between dietary acrylamide and cancer risk were performed in North European regions, where dietary exposure to acrylamide is higher than in the Eastern geographical regions (i.e., Japanese populations), which reported the lowest mean dietary exposure below 10 μg/day. Additionally, the no association found in this study may be explained by the lack of assessment of co-exposure to other compounds in food matrixes, including other foodborne carcinogens (62) or substances that can confer protection to carcinogenic compounds (63, 64).

The findings related to the slightly increased risk of lymphomas in relation to high dietary acrylamide exposure restricted to the Western geographical region needs to be interpreted with caution as they are based on one single study (16) and combining the subtypes of lymphoma, characterized by very different clinical features, as well as genetic profile (65). These findings disagree to some extent with those reported by Pelucchi et al. (46). However, the latter did not investigate lymphomas and multiple myeloma separately and considered the ATBC study that we excluded from the main analysis because based on smokers alone. The biological mechanisms underlying the potentially increased risk of lymphoma in relation to high dietary acrylamide exposure in Western regions are unclear, but explanations may be speculated. Firstly, the hydrophilic nature of acrylamide leads to systemic exposure and so all human tissues, including the hemopoietic and lymphoid tissues, are potential targets for acrylamide-induced carcinogenesis. Also, acrylamide has been shown to induce carcinogenicity through dysregulation of the endocrine system, including hormones (66) and this has been suggested to play a role in the pathophysiology of lymphomas (16). The restriction of these findings to the Western geographical areas may be attributed to possible interactions between genetic and environmental exposures determinants, such as different sources of dietary acrylamide (67, 68).

In general, the similar results found in smokers and never smokers in our meta-analysis are in line with a previous meta-analysis (46). The results of increased risk of lung cancer in smokers and melanoma in non-smokers need be carefully interpreted since they are based on few and heterogenous studies. Also, for some of the cancers considered, the summary estimates suggested that never smokers could experience a slightly positive association between dietary acrylamide and cancer risk, which was not detectable in smokers. These results are in line with previous studies investigating this relationship by smoking status (16, 60). A possible explanation is that for the same level of exposure, less dietary acrylamide is converted to the genotoxic and carcinogenic metabolite of acrylamide, glycidamide, because smoking affects the metabolism of acrylamide by e.g., saturating enzymes involved in its conversion to glycidamide (55).

### Strengths and Limitations

Firstly, we have performed an updated meta-analysis of the relationship between dietary acrylamide and the risk of several site-specific cancer. Compared to the previous meta-analyses, we were able to add some studies for several site-specific cancers, resulting in summary estimates with higher precision and accuracy.

Secondly, we also investigated the shape of the relationship between acrylamide and site-specific cancer and we evaluated heterogeneity among some groups (types of cancer, smoking status, and geographical regions). However, for some outcomes, the results were still based on a very limited number of available studies.

Our systematic review and meta-analysis have some limitations. They are based on epidemiological studies that assessed dietary acrylamide exposure through self-reported questionnaires. Hence, we cannot exclude that the summary estimates may be affected by misclassification of exposure related to the self-reported dietary intake (2, 69). Moreover, the acrylamide food database used in the separate studies, will not fully capture the variations in acrylamide levels between brands of a given food and in different food categories, as well as the different cooking methods used at home by the participants. However, results from a validation study showed a significant correlation between dietary acrylamide exposure and the acrylamide hemoglobin adducts (70). Furthermore, results from the only study investigating the exposure to acrylamide measured by acrylamide hemoglobin adducts in relation to cancer (i.e., prostate) showed no association in a Swedish population (20). In addition, the low number of available studies for each the site-specific cancer was not compatible with subgroup analyses and, so, we could not conduct a sensitivity analysis stratified by type of study design (case-control and cohort studies), which may have led to different summary estimates and, consequently, different conclusions. However, since there is little evidence for the occurrence of reverse causation or other biases in the studies with case-control design, the potential source of bias is very limited.

Additionally, for several of the studies included in the analysis stratified by smoking, doses of acrylamide by smoking status were not available and we used the doses indicated for the general population. Hence, the results on possible differences between smokers and non-smokers need to be carefully interpreted.

## Conclusion

Based on the results of this dose-response meta-analysis of epidemiological studies, higher dietary acrylamide exposure (vs. lower) was not associated with an increased risk of several site-specific cancers. If associations between dietary acrylamide and the risk of several site-specific cancers were present, the shape of the dose-response relationships would be with no thresholds. Smoking status might modify the relation between dietary acrylamide and some site-specific cancers, but findings need to be furtherly investigated.

## Data Availability Statement

The original contributions presented in the study are included in the article/Supplementary Material, further inquiries can be directed to the corresponding author.

## Author Contributions

TF, TH, AV, and FL conceptualized the study and drafted the manuscript. TF performed the statistical analyses. All authors contributed substantively to this work, involved in the interpretation of results, editing of the manuscript, and approved manuscript submission.

## Conflict of Interest

The authors declare that the research was conducted in the absence of any commercial or financial relationships that could be construed as a potential conflict of interest.

## Publisher’s Note

All claims expressed in this article are solely those of the authors and do not necessarily represent those of their affiliated organizations, or those of the publisher, the editors and the reviewers. Any product that may be evaluated in this article, or claim that may be made by its manufacturer, is not guaranteed or endorsed by the publisher.

## Abbreviations

ATBC, Alpha-Tocopherol: Beta-Carotene Cancer
CI: Confidence Interval
DNA: Deoxyribonucleic acid
HR: Hazard Ratio
IARC: International Agency for Research on Cancer
MeSH: Medical Subject Headings
MOOSE: Meta-analysis Of Observational Studies in Epidemiology
OR: Odds Ratio
PRISMA: Preferred Reporting Items for Systematic Reviews and Meta-Analyses
ROBINS-I: Risk of Bias In Non-randomized Studies of Interventions
RR: Relative Risk.
