# Supplementary material for: Dietary Acrylamide Exposure and Risk of Site-Specific Cancer: A Systematic Review and Dose-Response Meta-Analysis of Epidemiological Studies
Source: Front Nutr. 2022 Apr 25;9:875607. doi: 10.3389/fnut.2022.875607 (PMC9082595; doi:10.3389/fnut.2022.875607)

Appendix 1

Table S1. Search strategy for studies aimed to evaluate the association between dietary acrylamide and site-specific cancer. Details are provided below for PubMed, Web of Science and Scopus databases, though with database-specific adjustments accommodated to the requirements of each database as follows.

**Review questions**<br>1) In the pooled analysis of all available and eligible studies, is dietary acrylamide exposure associated with risk of site-specific cancer?<br>2) In the pooled analysis of all available and eligible studies, what is the shape of the dose-response relationship between dietary acrylamide exposure and the risk of site-specific cancer?	
The search was conducted on March 7, 2022.<br>No time limit defined.<br>Language: English<br>Population: Adults or older adults (18 and over)<br>Intervention/Exposure: Higher levels of intake to dietary acrylamide<br>Comparator: Lower levels of intake to dietary acrylamide<br>Outcomes: Risk of cancer incidence (RR; HR or OR)<br>Study design: Cohort studies, case-cohort and case-control studies<br><br>Exclusion criteria: breast, endometrial and ovarian carcinoma risk	
**Database**	**Searches**
PubMed	((acrylamides[MeSH Terms]) OR (acrylamide*[Title/Abstract])) AND (((((((Neoplasms[MeSH Terms]) OR (Neoplasia*[Title/Abstract])) OR (Neoplasm*[Title/Abstract])) OR (Tumor*[Title/Abstract])) OR (Cancer*[Title/Abstract])) OR (Malignanc*[Title/Abstract])) AND ((Risk[MeSH Terms]) OR (Risk*[Title/Abstract])))
Web of Science	(TS=(Neoplasia*) OR TS=(Neoplasm*) OR TS=(Tumor*) OR TS=(Cancer*) OR TS=(Malignanc*)) AND TS=(risk*) AND TS=(acrylamide*)
Scopus	(TITLE-ABS-KEY (neoplasia*) OR TITLE-ABS-KEY (neoplasm*) OR TITLE-ABS-KEY (tumor*) OR TITLE-ABS-KEY (cancer*) OR TITLE-ABS-KEY (malignanc*)) AND TITLE-ABS-KEY (risk*) AND TITLE-ABS-KEY (acrylamide*)

Appendix 2

Figure S1. Forest plot of dietary acrylamide exposure and risk of oral cavity cancer. CI: confidence interval; RR: relative risk.

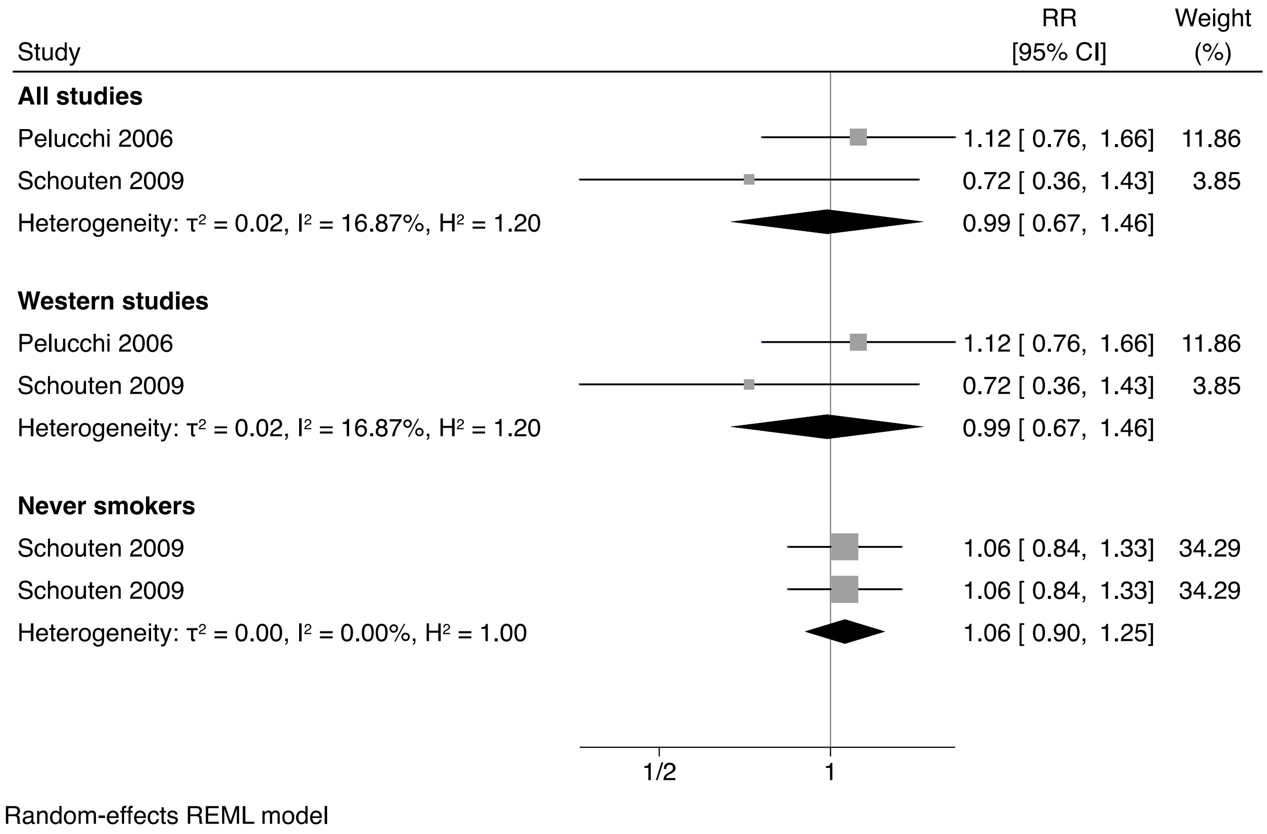

Figure S2. Forest plot and funnel plot of dietary acrylamide exposure and risk of esophageal cancer. CI: confidence interval; RR: relative risk; SE: standard error.

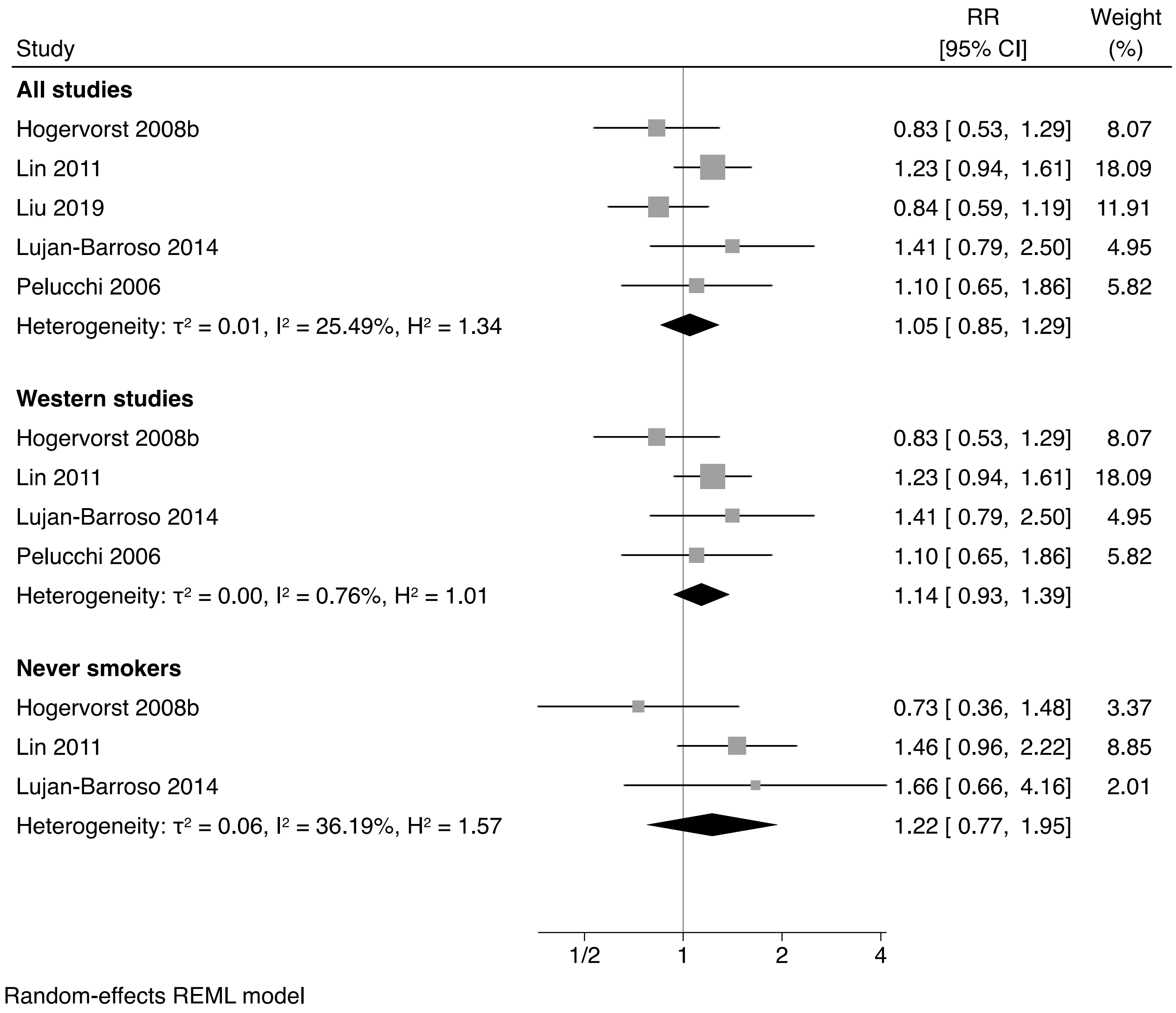

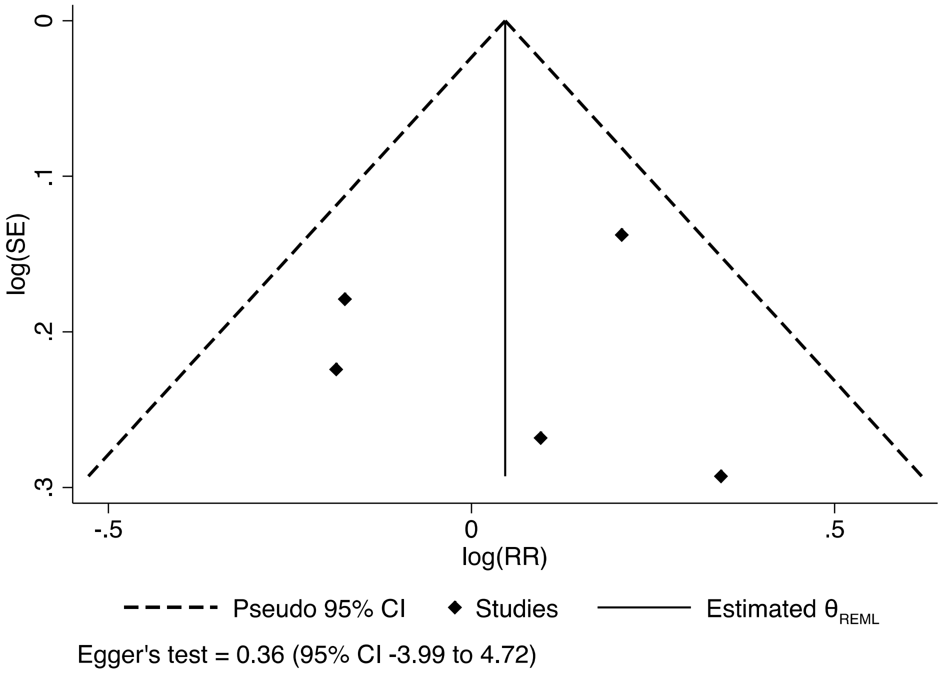

Figure S3. Forest plot of dietary acrylamide exposure and risk of stomach cancer. CI: confidence interval; RR: relative risk.

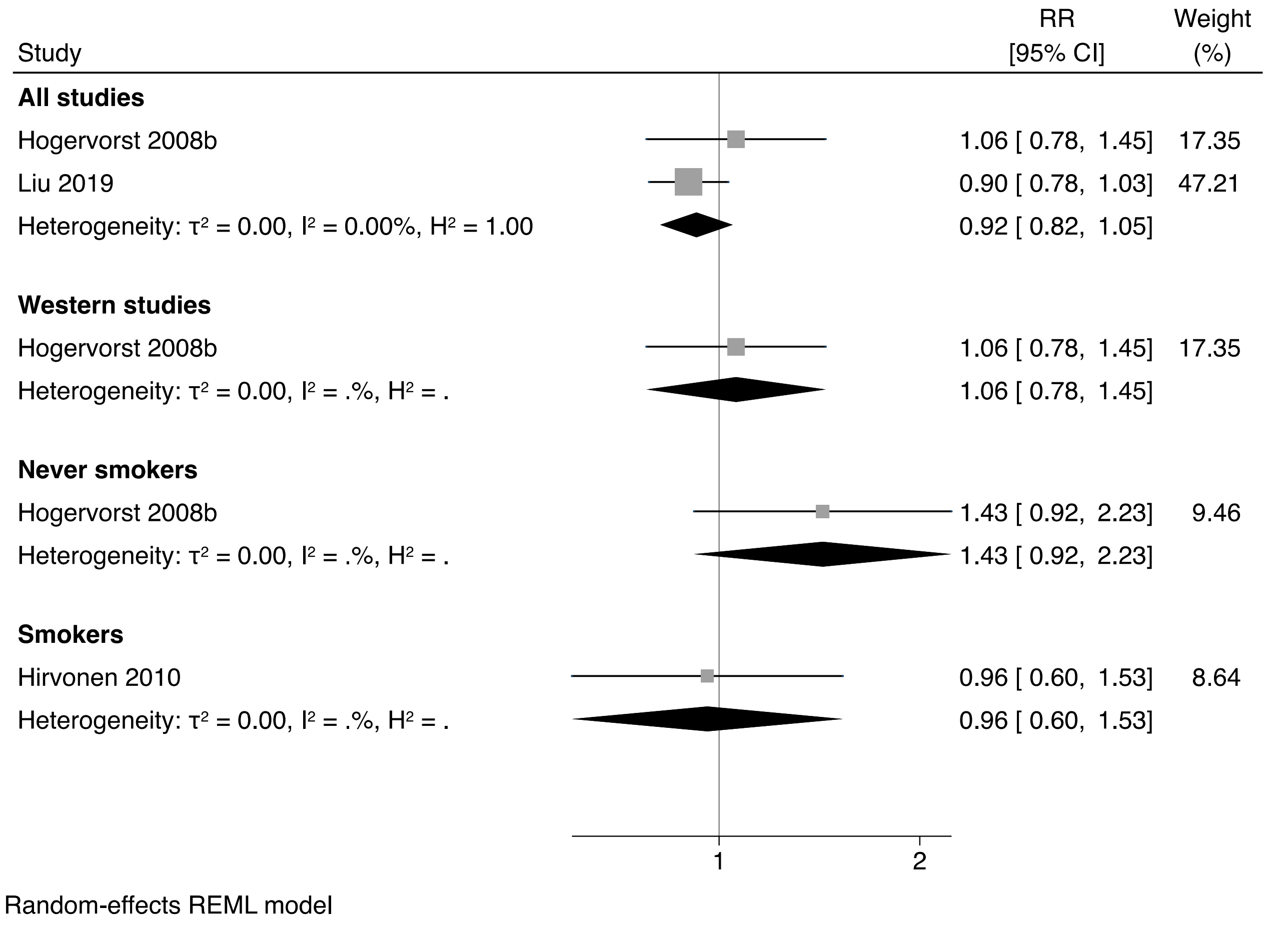

Figure S4. Forest plot and funnel plot of dietary acrylamide exposure and risk of colorectal cancer. CI: confidence interval; RR: relative risk; SE: standard error.

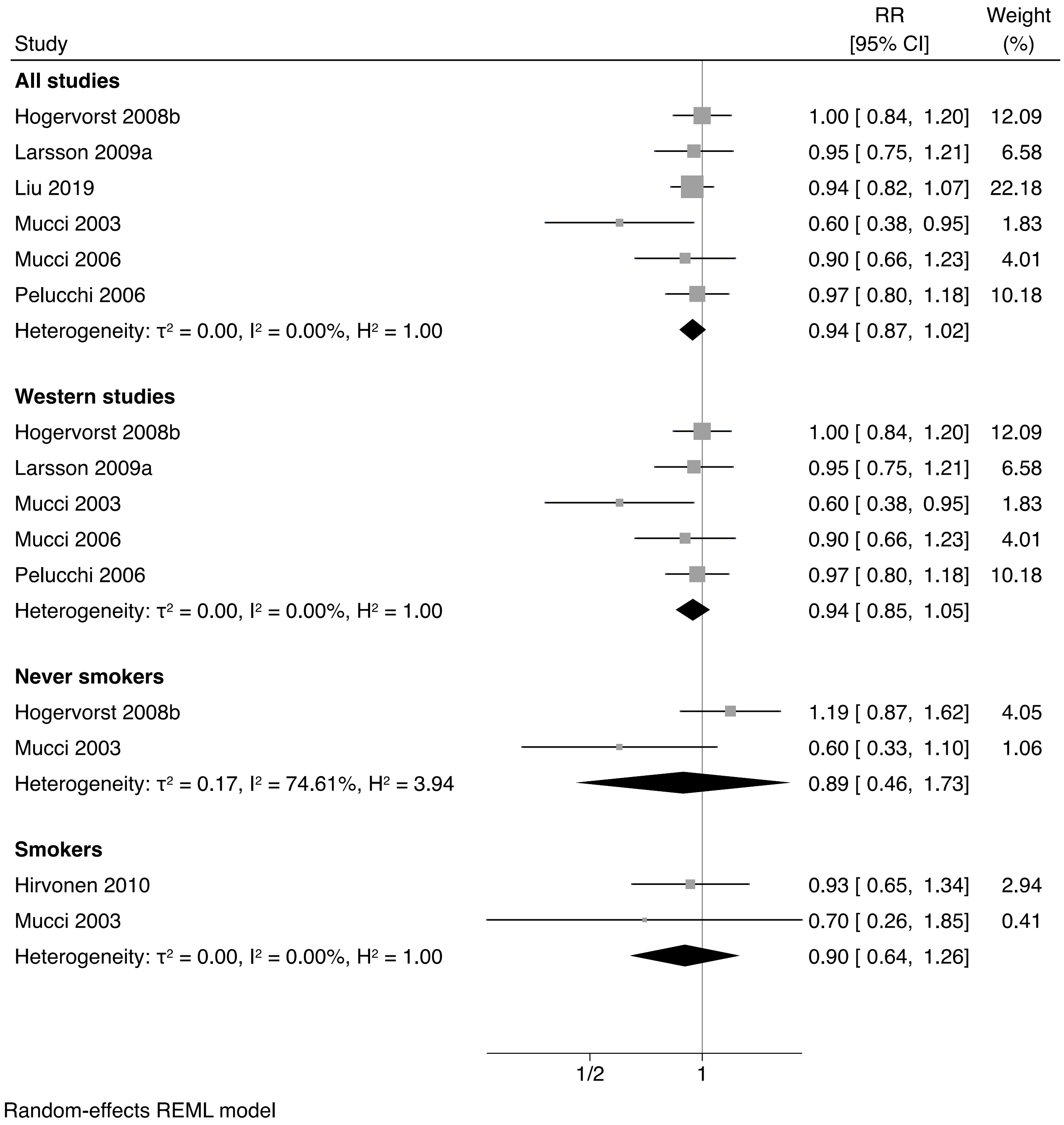

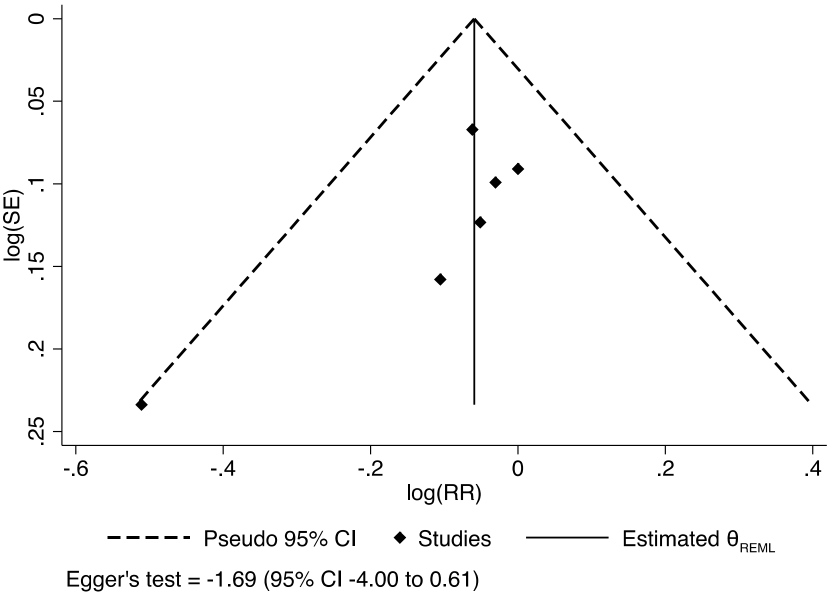
0
.05
.1
.15
.2
.25
log(SE)
-.6
-.4
-.2
0
.2
.4
log(RR)
Pseudo 95% CI
Studies
Estimated θ REML
Egger's test = -1.69 (95% CI -4.00 to 0.61)

Figure S5. Forest plot of dietary acrylamide exposure and risk of colon cancer. CI: confidence interval; RR: relative risk.

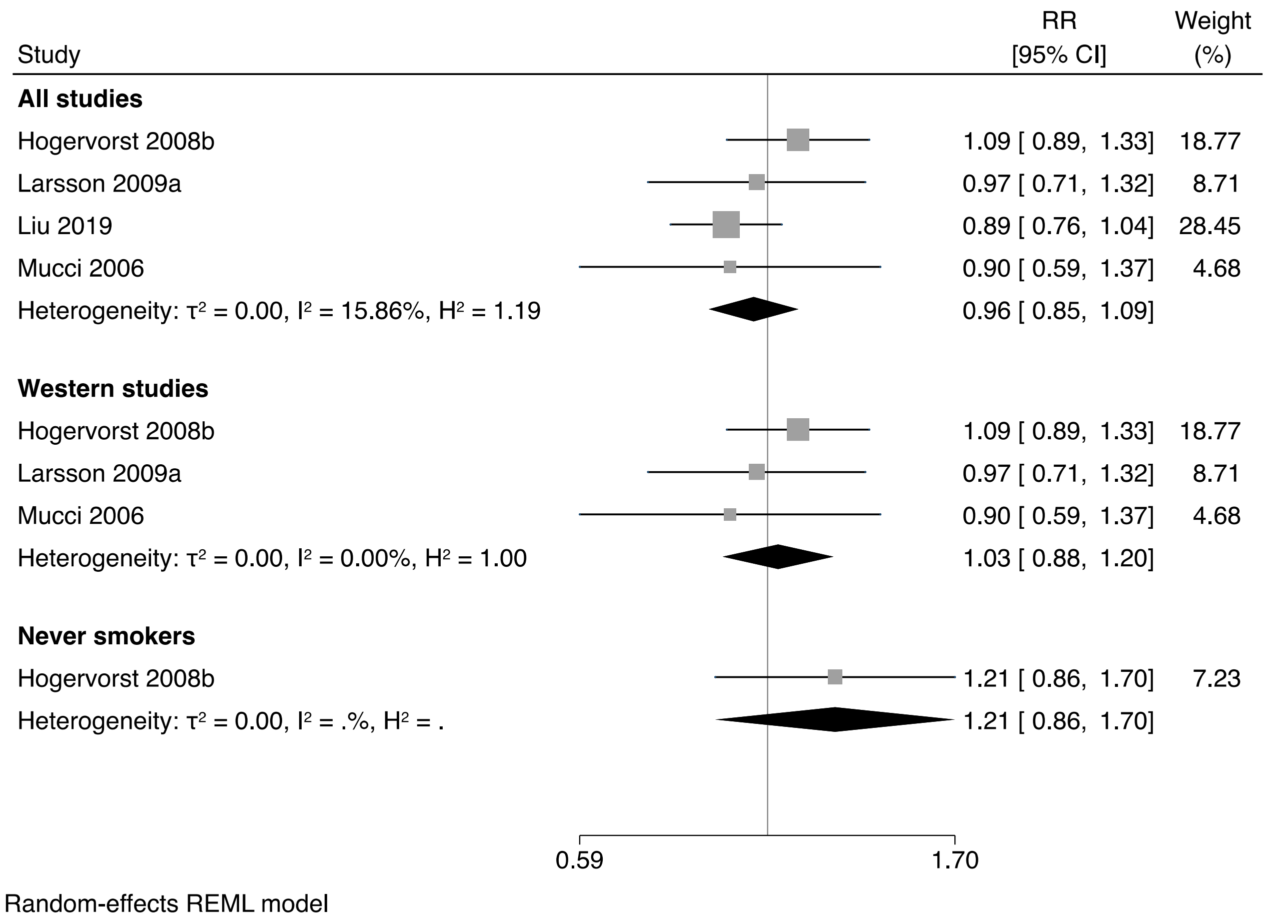

Figure S6. Forest plot of dietary acrylamide exposure and risk of rectal cancer. CI: confidence interval; RR: relative risk.

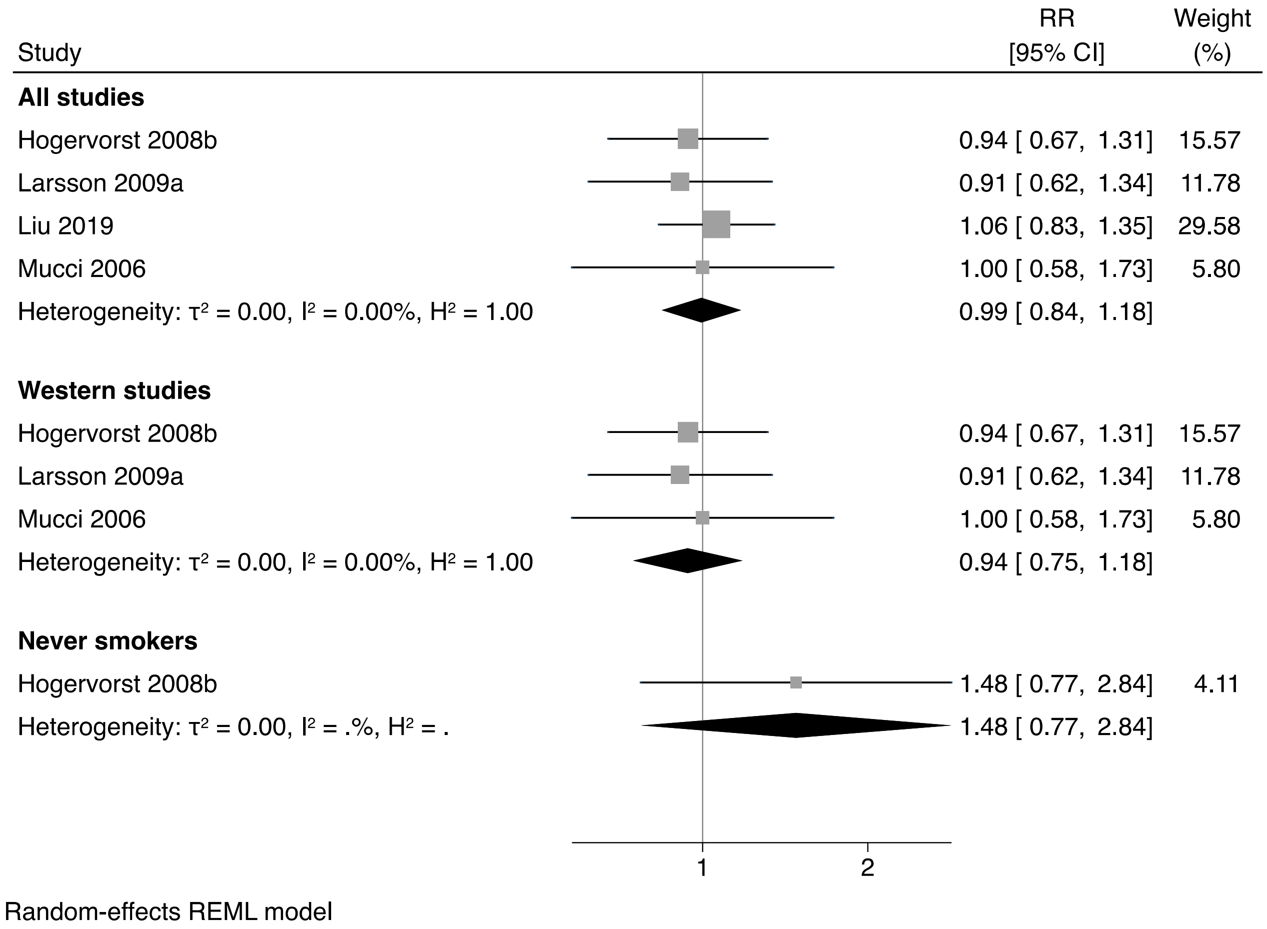

Figure S7. Forest plot and funnel plot of dietary acrylamide exposure and risk of pancreatic cancer. CI: confidence interval; RR: relative risk; SE: standard error.

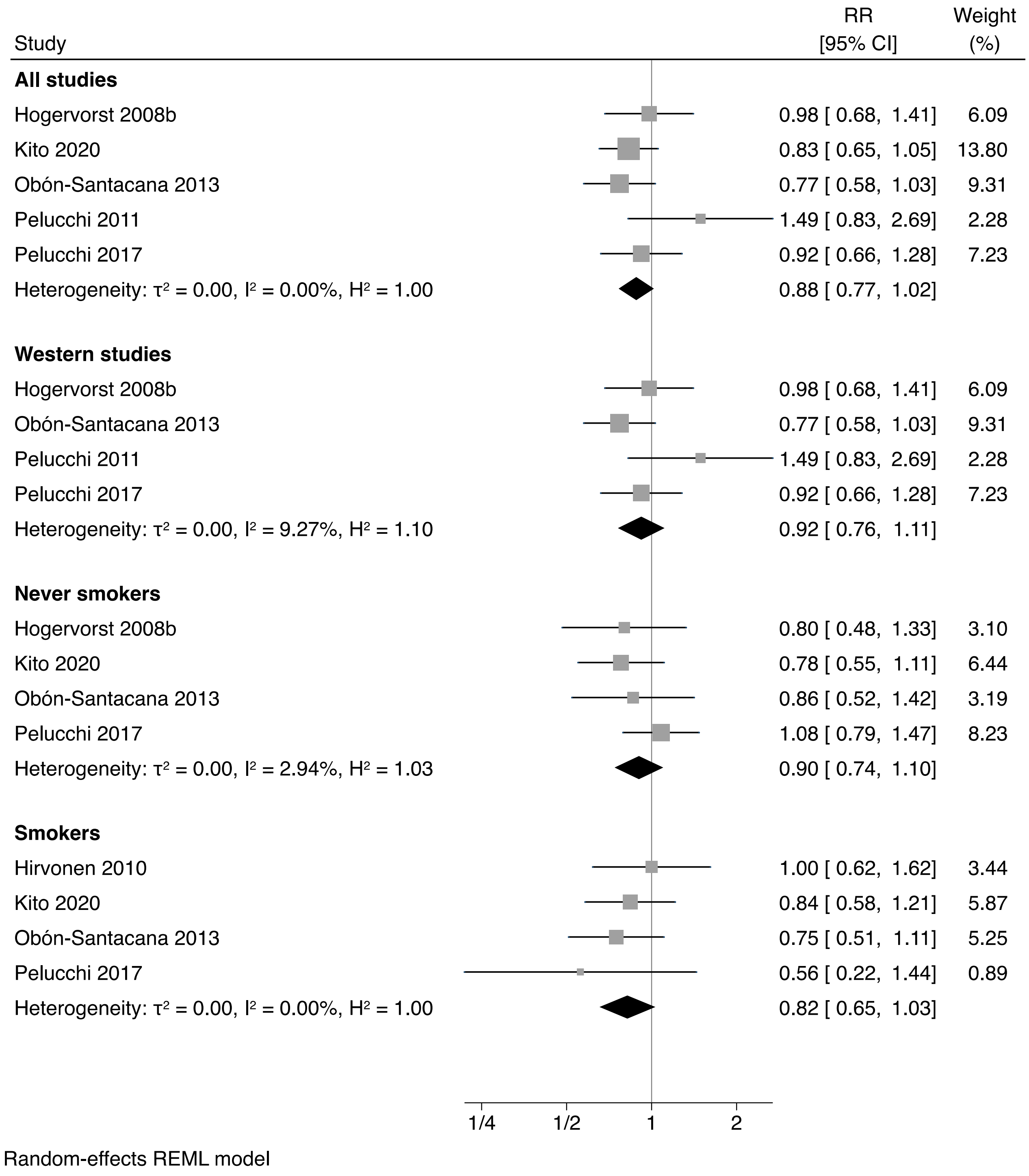

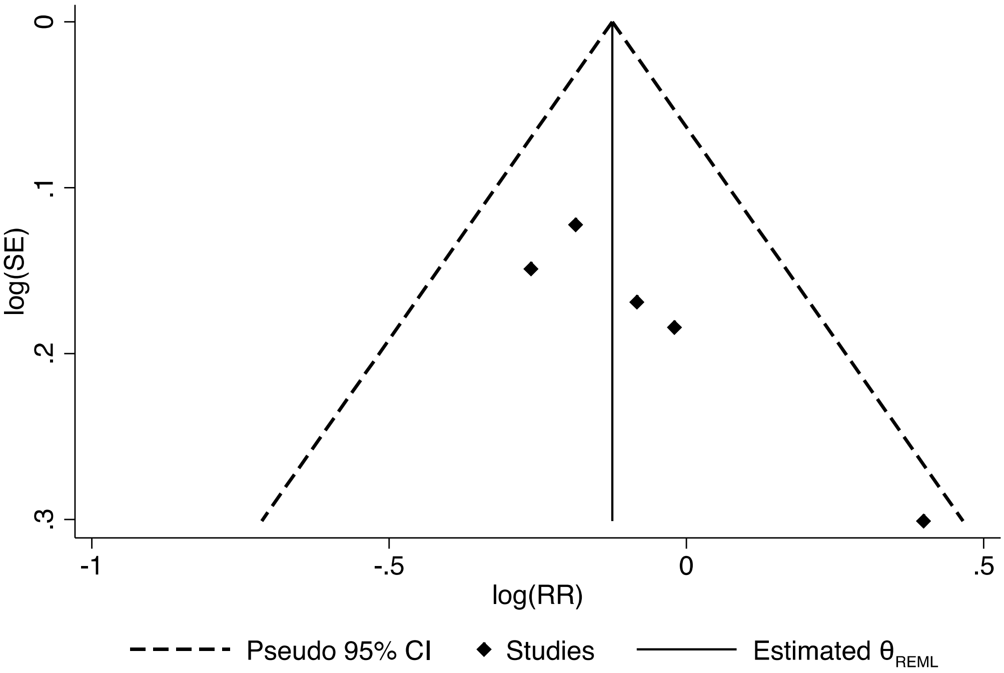

Egger's test = 3.32 (95% CI -0.08 to 6.73)

Figure S8. Forest plot of dietary acrylamide exposure and risk of laryngeal cancer. CI: confidence interval; RR: relative risk.

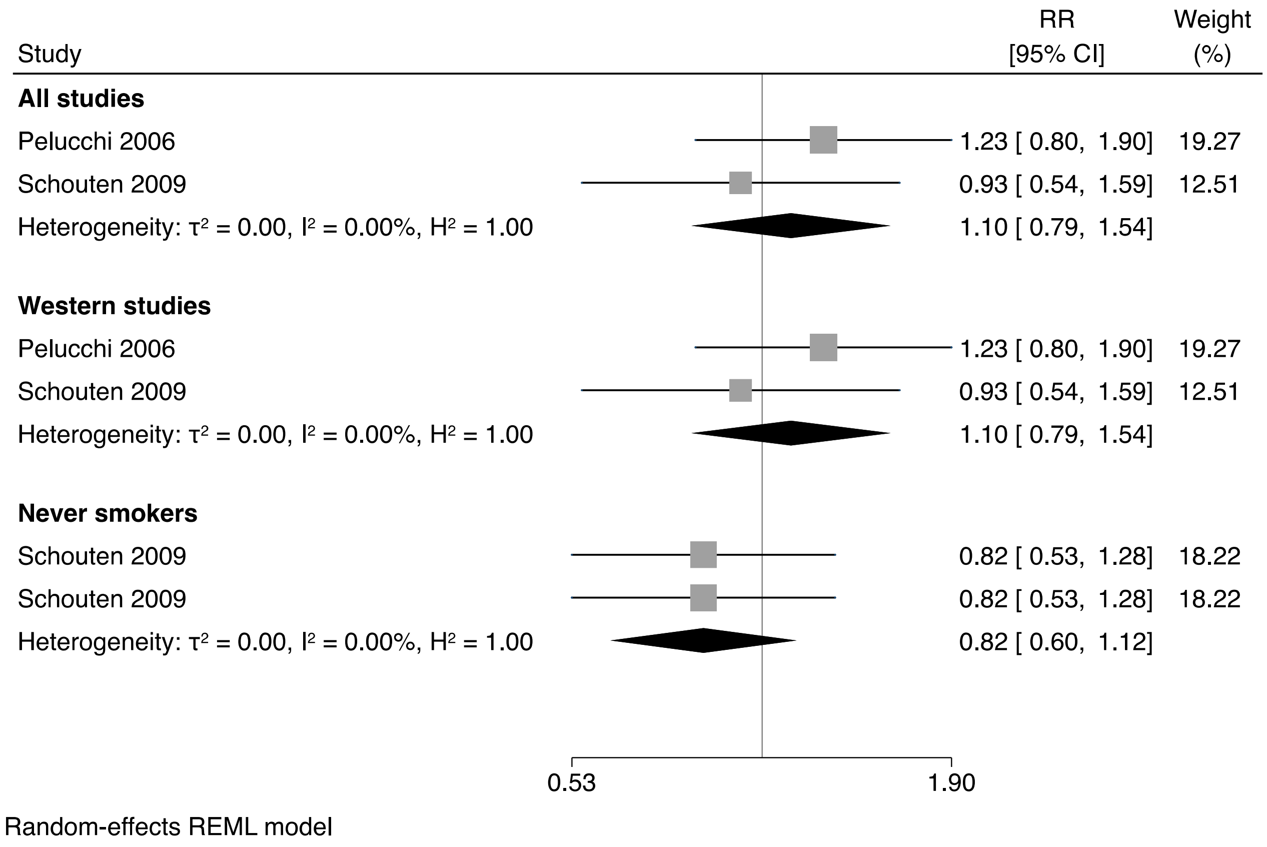

Figure S9. Forest plot of dietary acrylamide exposure and risk of lung cancer. CI: confidence interval; RR: relative risk.

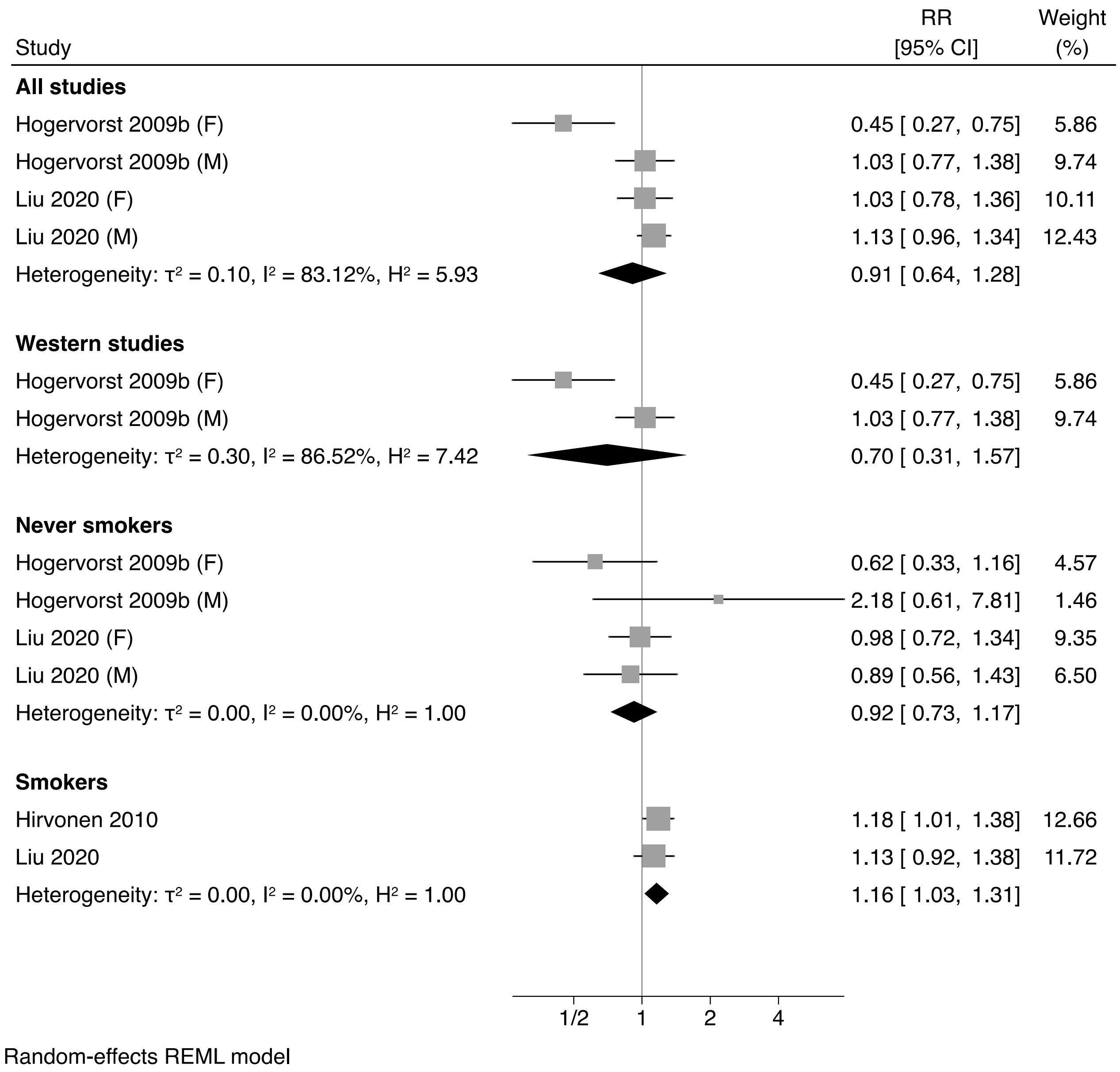

Figure S10. Forest plot of dietary acrylamide exposure and risk of bladder cancer. CI: confidence interval; RR: relative risk.

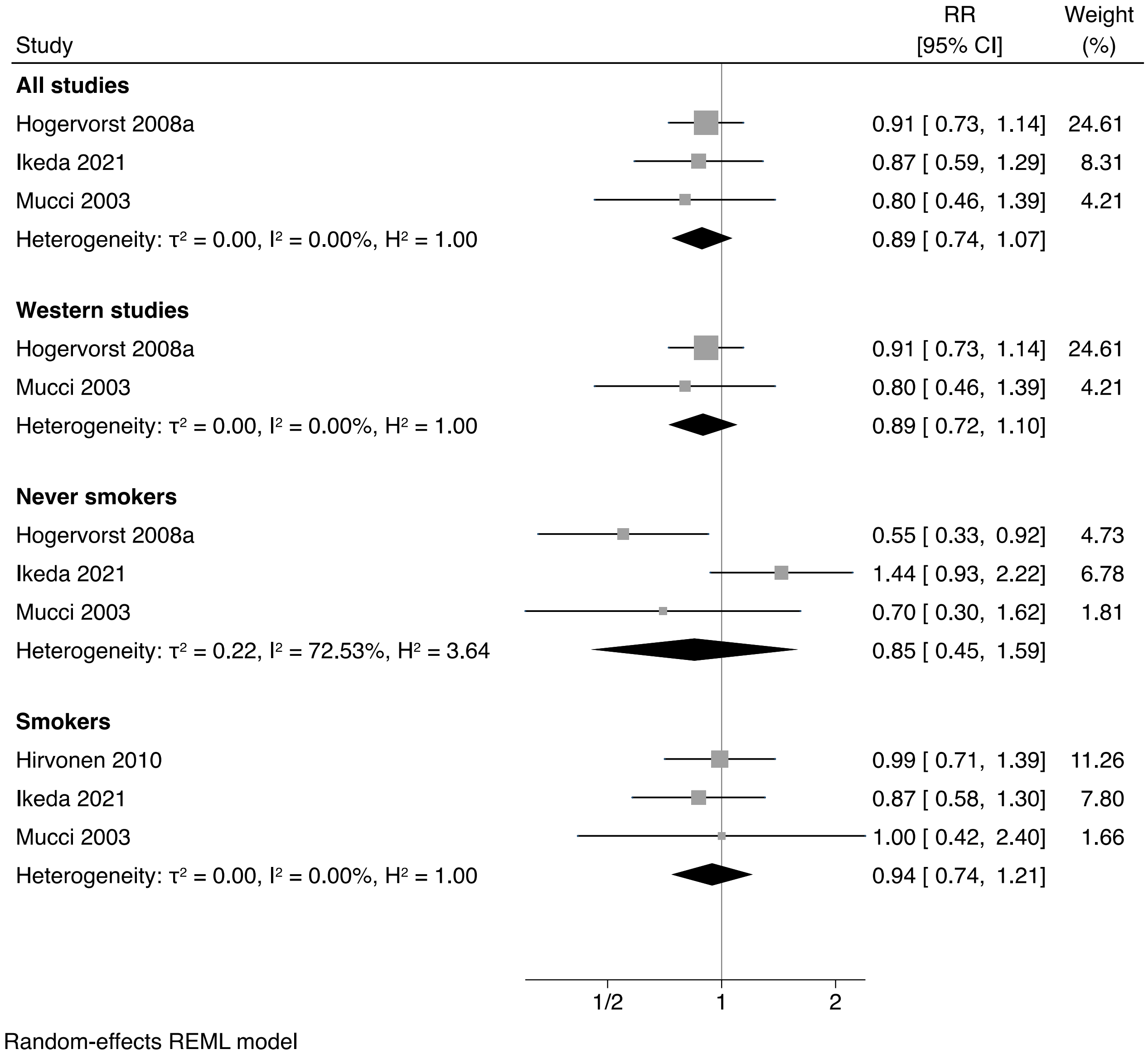

Figure S11. Forest plot and funnel plot of dietary acrylamide exposure and risk of renal cancer. CI: confidence interval; RR: relative risk; SE: standard error.

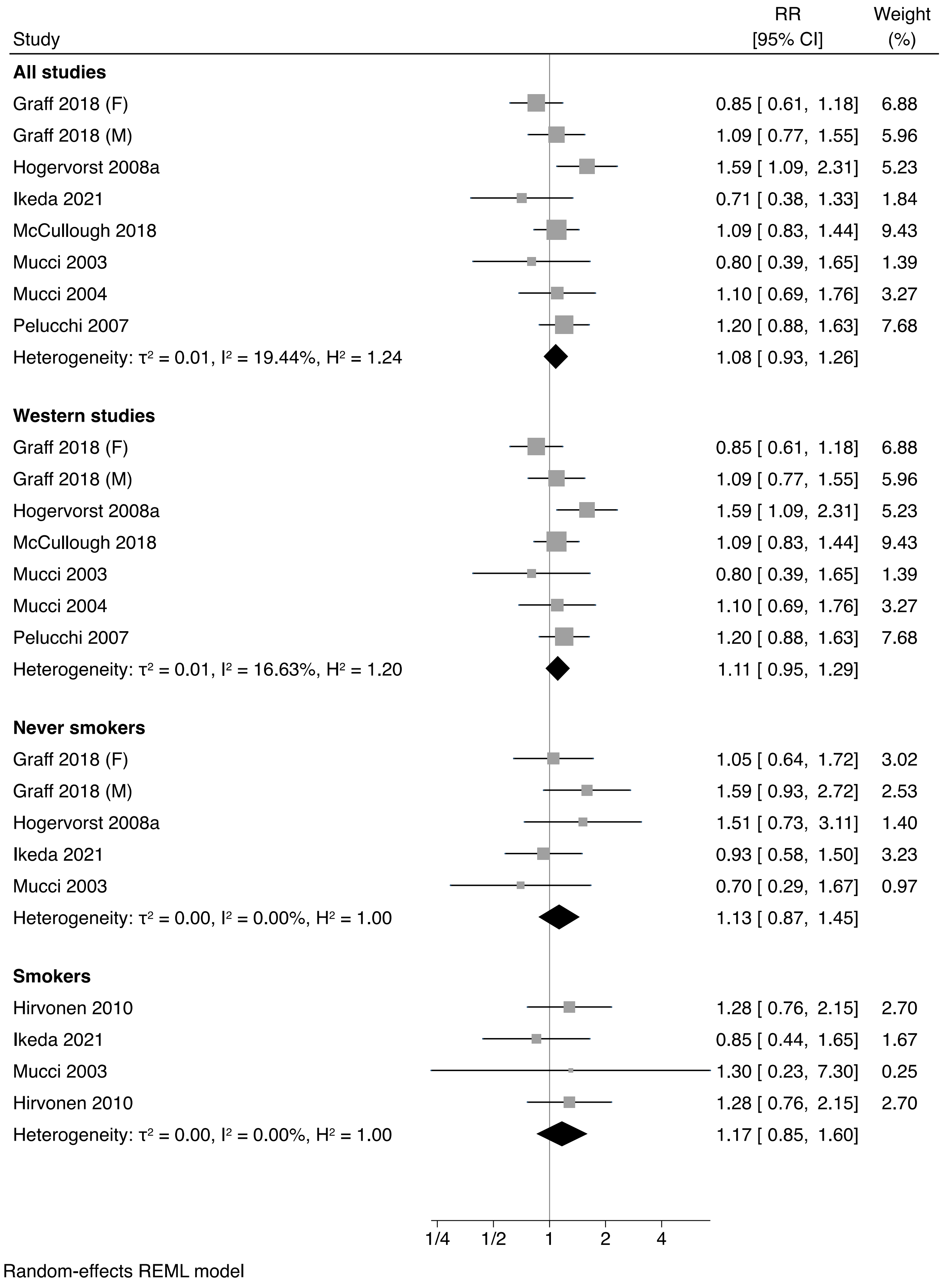

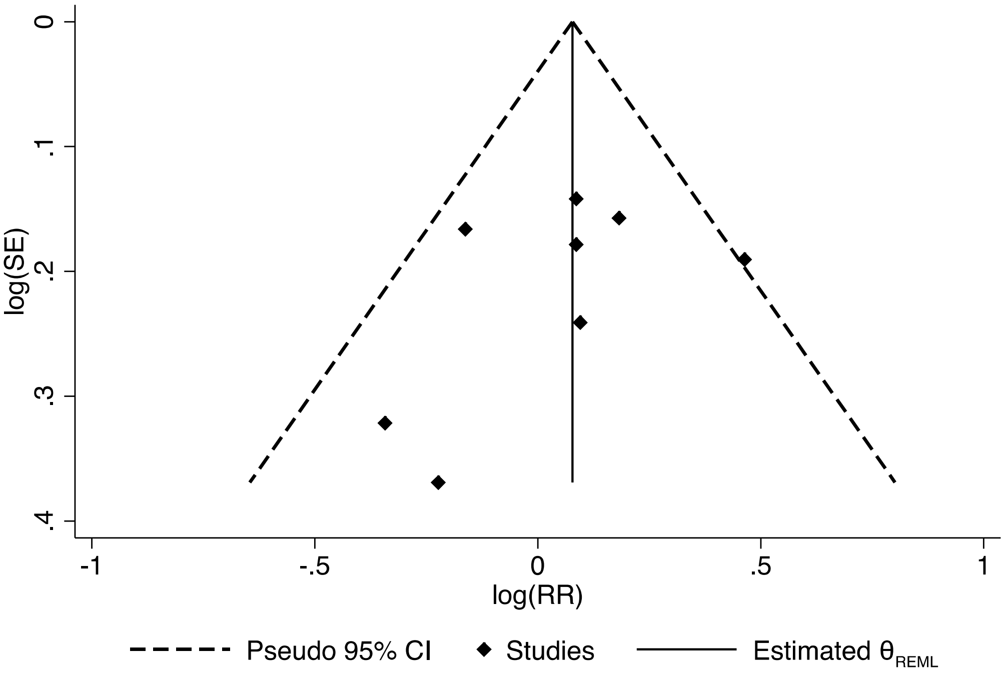

Egger's test = -1.52 (95% CI -4.29 to 1.24)

Figure S12. Forest plot and funnel plot of dietary acrylamide exposure and risk of prostatic cancer. CI: confidence interval; RR: relative risk; SE: standard error.

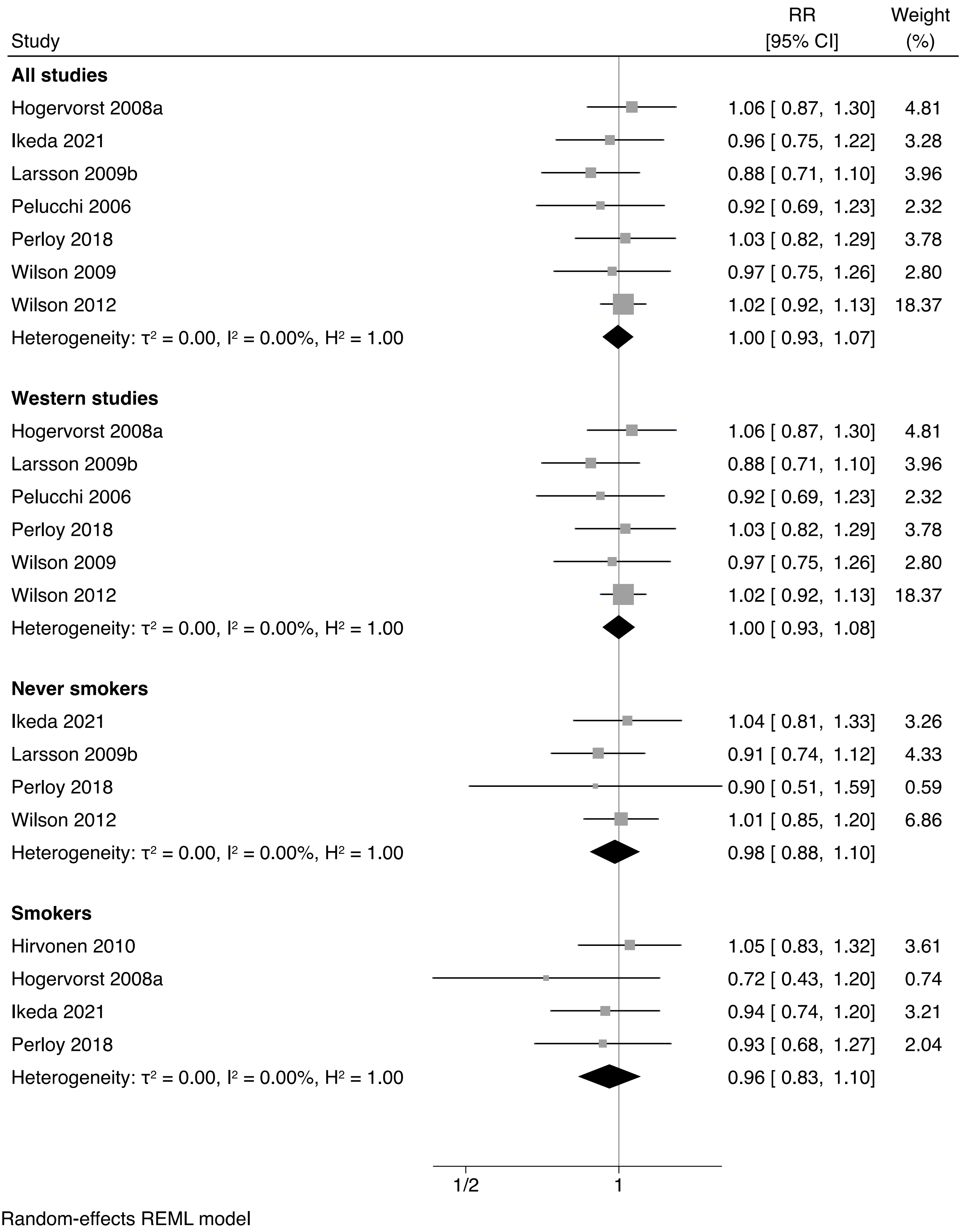

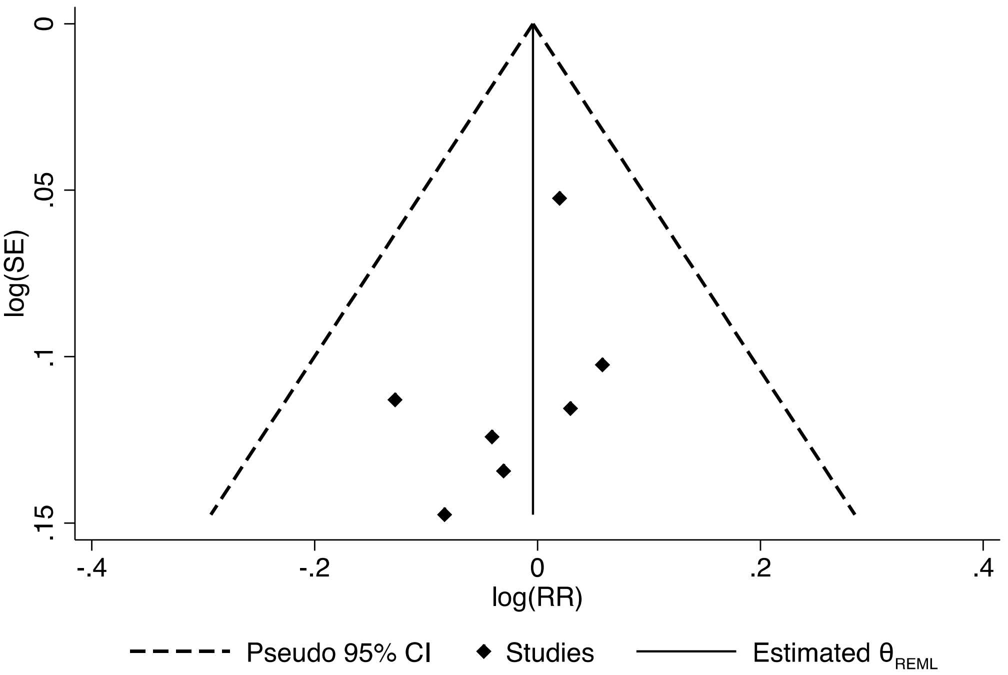

Egger's test = -0.81 (95% CI -2.84 to 1.23)

Figure S13. Forest plot of dietary acrylamide exposure and risk of lymphoma. CI: confidence interval; RR: relative risk.

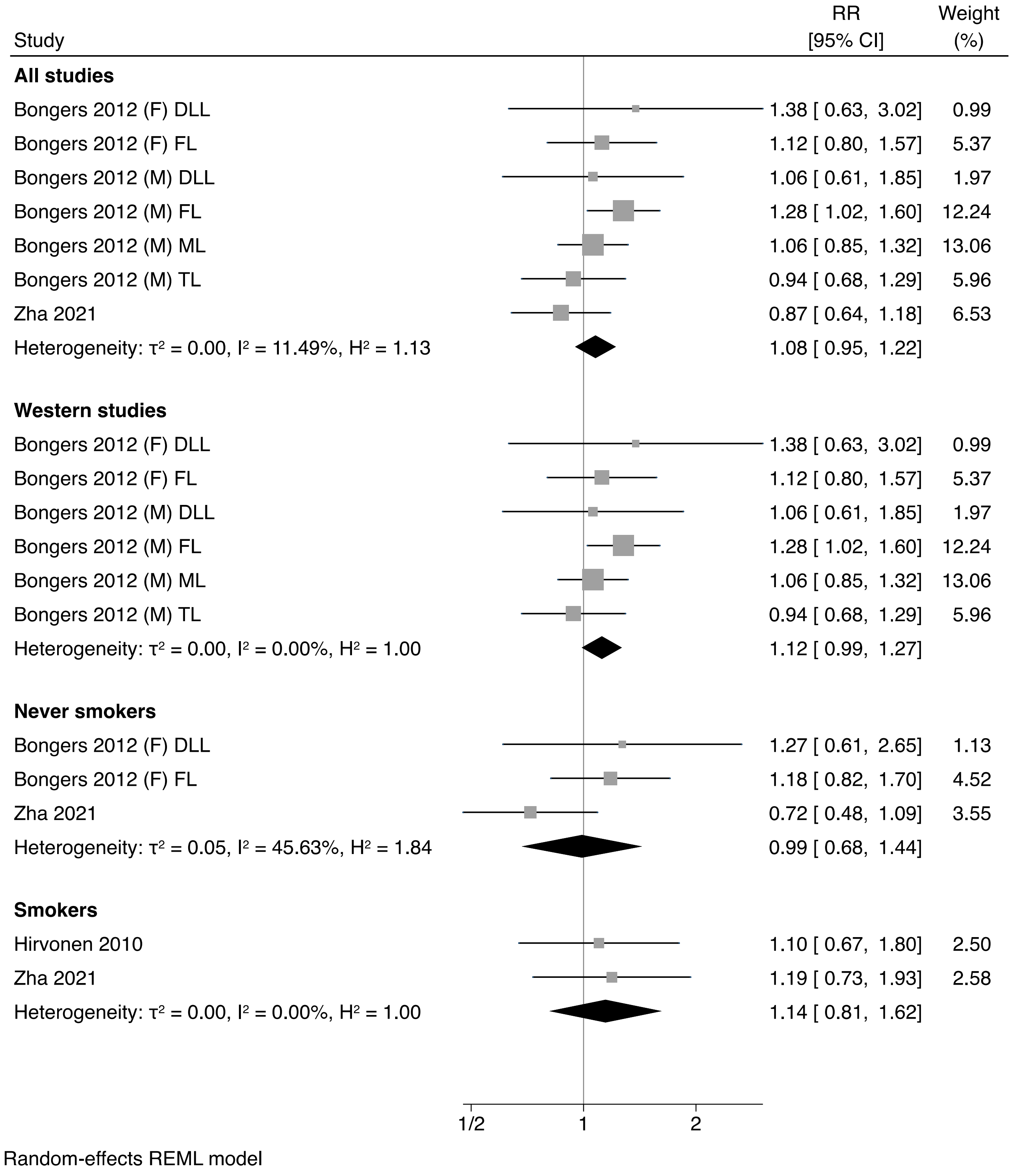

Figure S14. Forest plot of dietary acrylamide exposure and risk of multiple myeloma. CI: confidence interval; RR: relative risk.

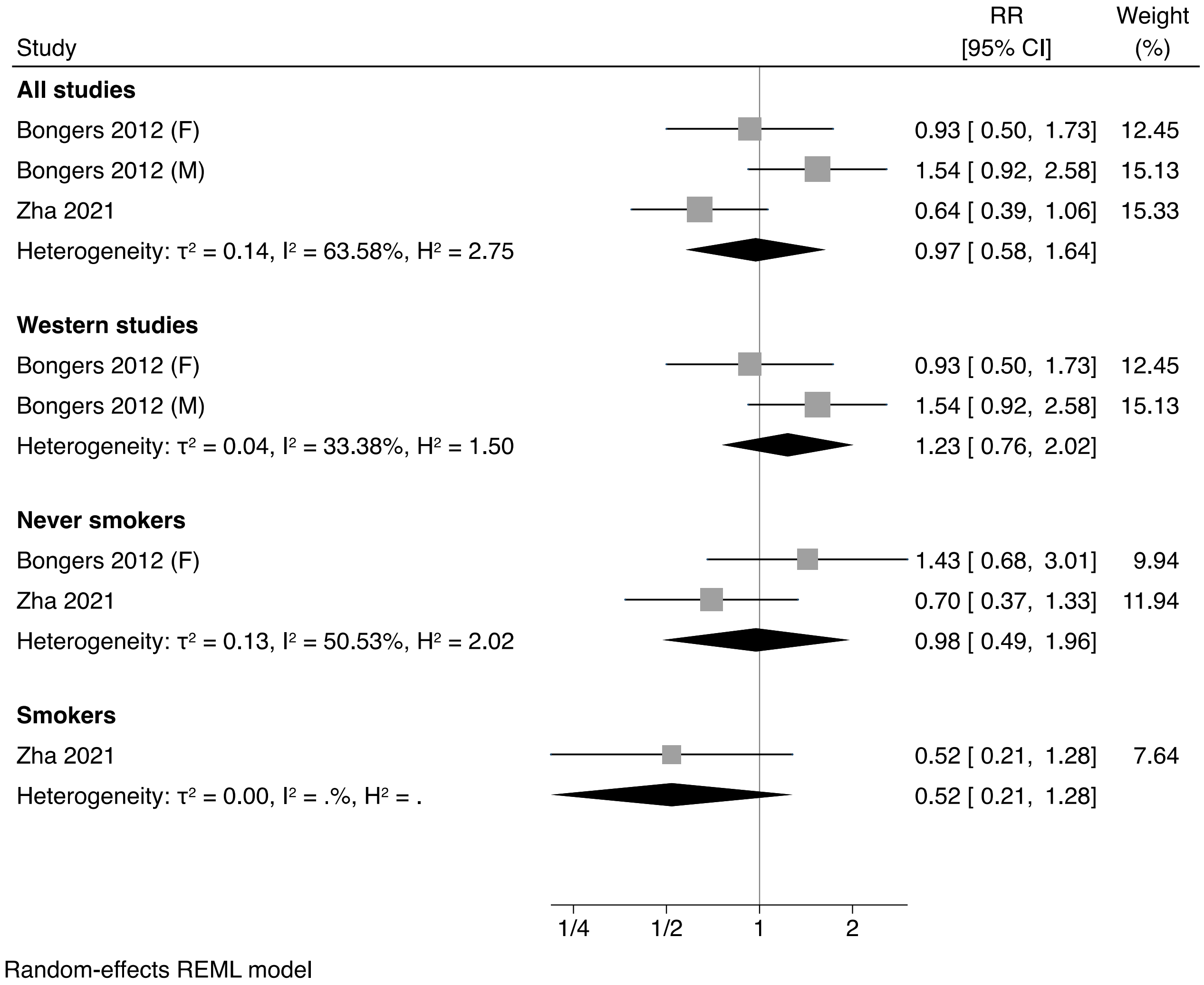

Figure S15. Forest plot of dietary acrylamide exposure and risk of melanoma. CI: confidence interval; RR: relative risk.

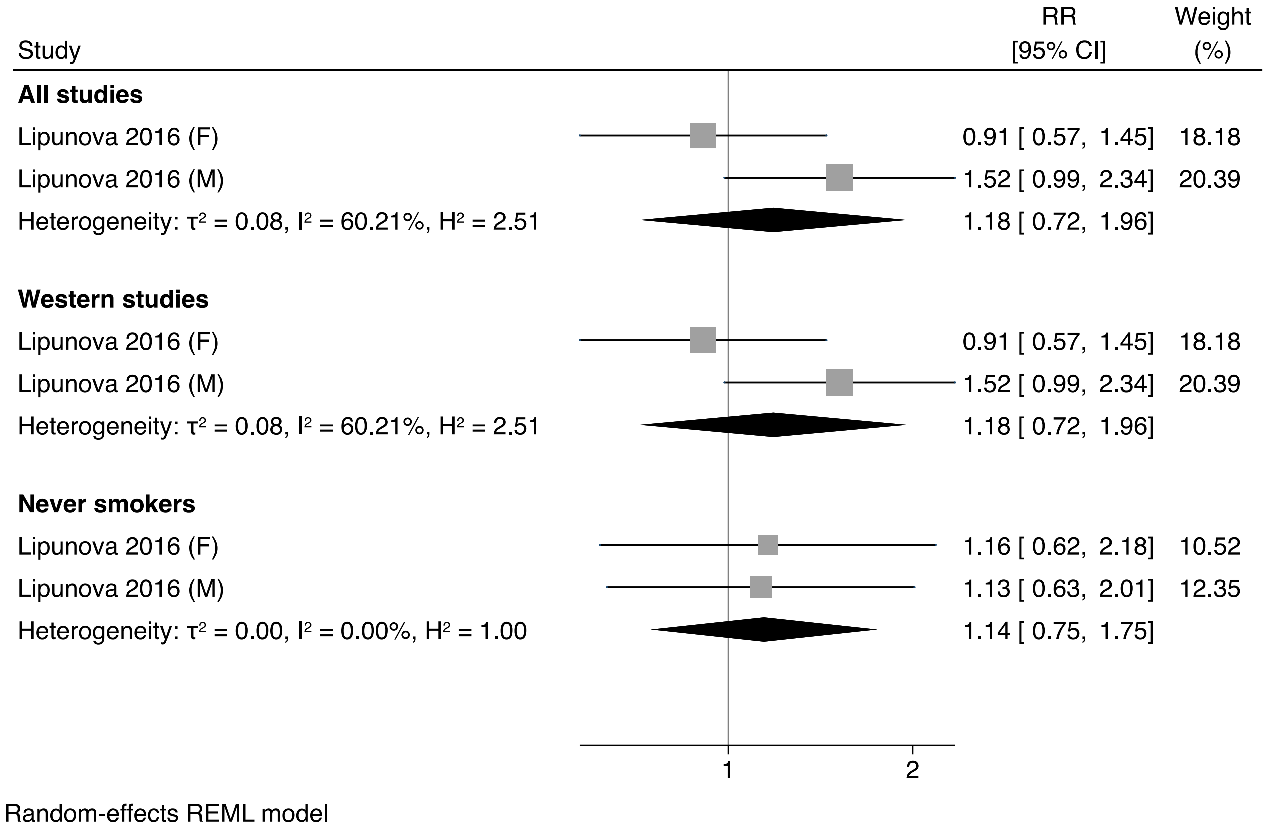

Figure S16. Results of the risk of bias assessment, using the ROBINS-I. Risk of bias domains by studies included.

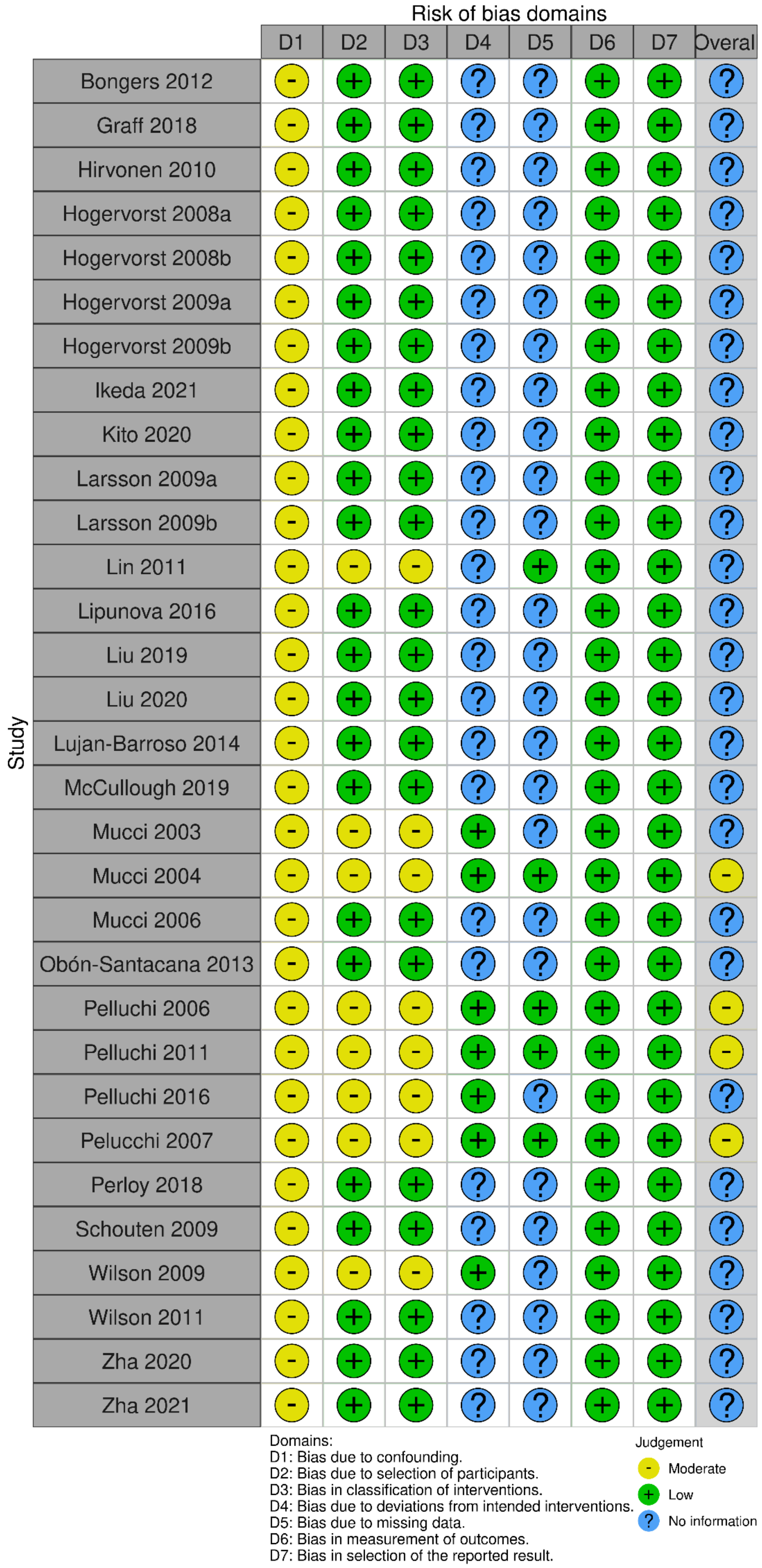

Figure S17. Results of the risk of bias assessment, using the ROBINS-I. Overall graphical representation of risk of bias by domains

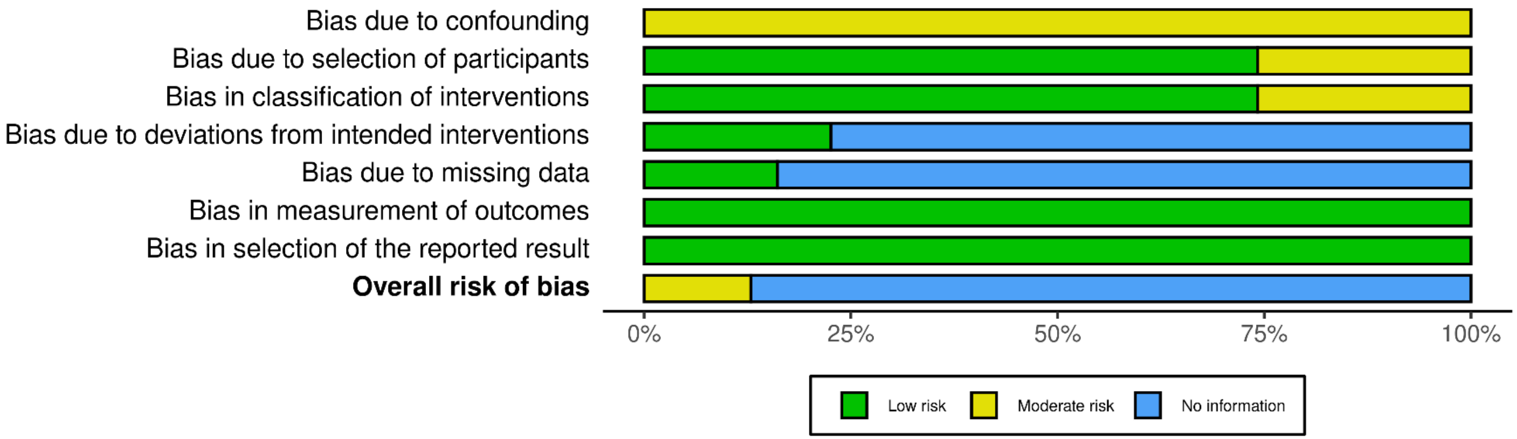

Supplement: Supplementary file 1 [file Data_Sheet_1.pdf]
